# Diversifying selection and climatic effects on major histocompatibility complex class II gene diversity in the greater horseshoe bat

**DOI:** 10.1111/eva.13528

**Published:** 2023-01-08

**Authors:** Xiaolin Li, Tong Liu, Aoqiang Li, Yanhong Xiao, Keping Sun, Jiang Feng

**Affiliations:** ^1^ Jilin Provincial Key Laboratory of Animal Resource Conservation and Utilization Northeast Normal University Changchun China; ^2^ Key Laboratory of Vegetation Ecology, Ministry of Education Changchun China; ^3^ College of Life Science, Jilin Agricultural University Changchun China

**Keywords:** bat, climatic effect, evolution, MHC, selection

## Abstract

Heterogeneous pathogenic stress can shape major histocompatibility complex (MHC) diversity by influencing the functional plasticity of the immune response. Therefore, MHC diversity could reflect environmental stress, demonstrating its importance in uncovering the mechanisms of adaptive genetic variation. In this study, we combined neutral microsatellite loci, an immune‐related MHC II‐DRB locus, and climatic factors to unravel the mechanisms affecting the diversity and genetic differentiation of MHC genes in the greater horseshoe bat (*Rhinolophus ferrumequinum*), a species with a wide geographical distribution that has three distinct genetic lineages in China. First, increased genetic differentiation at the MHC locus among populations compared using microsatellites indicated diversifying selection. Second, the genetic differentiation of MHC and microsatellites were significantly correlated, suggesting that demographic processes exist. However, MHC genetic differentiation was significantly correlated with geographical distance among populations, even after controlling for the neutral markers, suggesting a major effect of selection. Third, although the MHC genetic differentiation was larger than that for microsatellites, there was no significant difference in the genetic differentiation between the two markers among genetic lineages, indicating the effect of balancing selection. Fourth, combined with climatic factors, MHC diversity and supertypes showed significant correlations with temperature and precipitation, but not with the phylogeographic structure of *R. ferrumequinum*, suggesting an effect of local adaptation driven by climate on MHC diversity. Moreover, the number of MHC supertypes varied between populations and lineages, suggesting regional characteristics and support for local adaptation. Taken together, the results of our study provide insights into the adaptive evolutionary driving forces at different geographic scales in *R. ferrumequinum.* In addition, climate factors may have played a vital role in driving adaptive evolution in this species.

## 1 INTRODUCTION

Understanding factors that have altered biodiversity in the past may help us to predict how diversity will be affected by current and future environmental changes such as the global climate change that is now underway as a result of human use of fossil fuels (Futuyma, 2009). As the most fundamental level of biodiversity, intraspecific genetic variation could be employed to increase our understanding of how biodiversity changes (Adams & Hadly, 2013; Ellegren & Galtier, 2016; Yiming et al., 2021). Genetic variation can determine or affect the way a species interacts with other species and with the environment, and variation also is a decisive factor for a species to successfully respond to human disturbance. The degree of intraspecific genetic variation also determines the ability of a species to adapt to environmental changes (Ellegren & Galtier, 2016). However, these mechanisms cannot be measured by relying solely on traditional neutral markers. MHC genes are ideal candidates for studying adaptive evolutionary processes in natural populations, as their polymorphism is thought to affect the functional plasticity of immune responses against heterogeneous pathogenic pressures (Kaufman, 2018). This feature suggests the immune response's susceptibility to environmental stress and its importance in uncovering mechanisms of adaptive genetic variation necessary for the long‐term survival of species or populations (Meyers & Bull, 2002; Van Tienderen et al., 2002).

The patterns of selection on MHC genes are simultaneously affected by multiple factors. In addition to mate choice selection and individual or kin recognition, MHC polymorphism is thought to be maintained primarily by non‐mutually exclusive pathogen‐mediated balancing selection (Spurgin & Richardson, 2010), including the heterozygote advantage hypothesis (Doherty & Zinkernagel, 1975), the rare‐allele advantage hypothesis (Slade & McCallum, 1992), and the fluctuating selection hypothesis (Hill, 1991). Moreover, MHC diversity could be associated with variation across environmental gradients and geographical scales, leading to diversifying selection (Hill, 1991; Spurgin & Richardson, 2010). Additionally, there is increasing evidence that in the MHC supertypes (ST), clusters of MHC variants grouped by functional properties, selection may act on functional MHC variants represented by MHC supertypes rather than on specific alleles (Sepil et al., 2013; Trachtenberg et al., 2003; Vlček et al., 2016). Furthermore, the role of selective pressures in shaping MHC genes could also be mediated by combined effects including demographic processes as well as behavioral and ecological differences in pathogen exposure (Baker et al., 2006; Moreno et al., 2016) because host‐pathogen interactions can be influenced by temperature, nutrient availability, and environmental stress (Björklund et al., 2015; Brunner & Eizaguirre, 2016). Ambient temperature and nutrient level changes can accelerate host local adaptation by affecting the selection of immune genes (Björklund et al., 2015; Brunner & Eizaguirre, 2016; Wegner et al., 2008), suggesting that environmental conditions play an important role in the adaptive evolution of species. However, only a few studies have focused on the environmental effects on MHC diversity; research has been conducted on striped mice (Froeschke & Sommer, 2014), hares (Awadi et al., 2018), golden jackals (Stefanović et al., 2021) and Atlantic salmon (Dionne et al., 2007).

Bats (Chiroptera), as a pool of pathogens, are transmitters of various pathogens and can adapt to diverse habitats (Chomel et al., 2015). This suggests that their immune system genes may undergo different evolutionary trajectories. To the best of our knowledge, previous researchers have focused more on MHC polymorphism and adaptive evolutionary processes of bats on relatively narrow geographical scales (Del Real‐Monroy et al., 2014; Richman et al., 2010; Salmier et al., 2016; Schad, Voigt, et al., 2012) rather than large geographical scales (Li et al., 2021; Qurkhuli et al., 2019). Other studies related to MHC genes have primarily focused on the relationships between pathogens and MHC genes (Schad, Dechmann, et al., 2012; Yi et al., 2020), while no study has considered the relationship between the environment and adaptive evolution of MHC genes in bats.

The greater horseshoe bat (*Rhinolophus ferrumequinum*) is a suitable species for elucidating the effects of various evolutionary forces on the MHC genes. First, *R. ferrumequinum* is widely distributed among different elevations and latitudes in China, suggesting that high habitat heterogeneity likely renders them susceptible to a diverse array of pathogens. Bats living at high latitudes face prolonged low temperatures in winter, while bats living at relatively low altitudes in temperate monsoon climates may experience relatively high temperatures in summer. In addition, the Qinling Mountains provide significant geographical isolation and limited gene flow between central and eastern populations of greater horseshoe bats (Flanders et al., 2009, 2011; Rossiter et al., 2002). Second, *R. ferrumequinum* can be divided into three genetic lineages: northeast (NE), central east (north of the Qinling Mountains, CE), and southwest (SW) based on neutral genetic markers (Sun et al., 2013). Those lineages have significantly different echolocation calls corresponding to their genetic differentiation pattern. Third, ecological selection rather than genetic drift has been associated with acoustic variation among different geographic populations of *R. ferrumequinum* (Sun et al., 2013), suggesting potential adaptive evolutionary processes. However, adaptive genetic markers have not been used in previous research. Considering the characteristic variation potentially related to environmental conditions, *R. ferrumequinum* is suitable for studying natural selection and adaptation in MHC genes.

Thus, in this study, we first investigated the variation of MHC II‐DRB genetic diversity and genetic structure in different populations within the three lineages of *R. ferrumequinum* distributed among heterogeneous environments. Second, we used several approaches to evaluate the effect of historical positive selection on each codon of the obtained sequences. Third, due to neutral processes having approximately equal effects on neutral loci, while the effects of natural selection vary between neutral and non‐neutral loci, we assessed the contribution of evolutionary forces between demographic processes and natural selection on MHC variation across spatial scales by comparing adaptive markers with neutral markers. Finally, we evaluated the effect of climate factors on MHC polymorphism and genetic structure.

## 2 MATERIALS AND METHODS

### 2.1 Field sampling and DNA extraction

**FIGURE 1:**
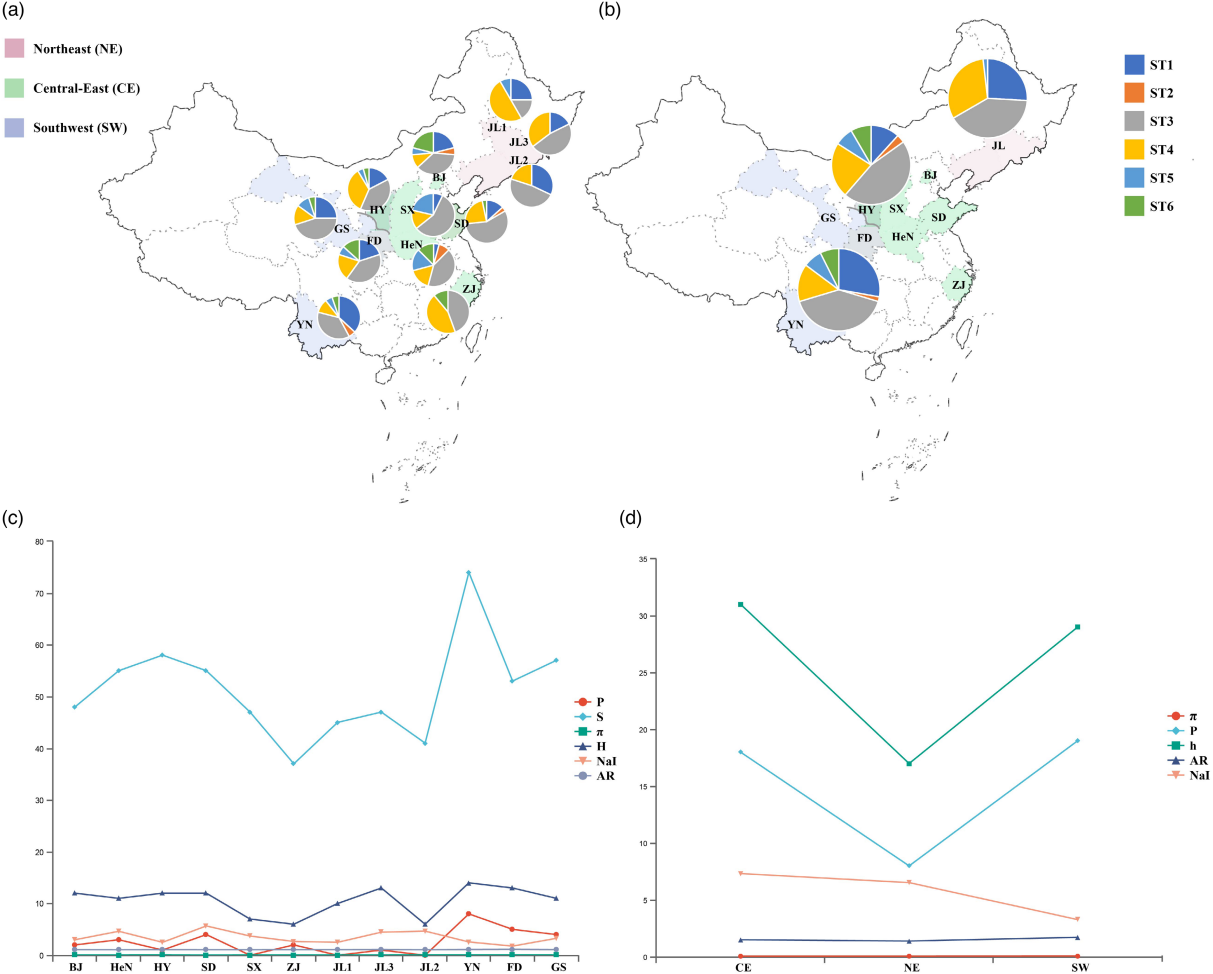
Distribution of *R. ferrumequinum* sampling sites and the supertype number in China. The supertype number is shown in the pie charts. Pie slice size is proportional to the number of supertypes. (a) Supertype number of populations. (b) Supertype number of genetic lineages and genetic diversity values for the MHC II‐*DRB* exon 2 loci in populations and genetic lineages of *R. ferrumequinum*. Genetic diversity values for MHC II‐DRB exon 2: P = private allele; S = number of segregating sites; π = average nucleotide diversity; h = number of haplotypes; NaI = mean number of alleles per individual; AR = allelic richness. MHC functional supertypes values: ST = number of supertypes; ST_SUM = total number of supertypes. (c) Genetic diversity of populations. (d) Genetic diversity of genetic lineages.

For MHC sequencing, a total of 121 individuals were sampled between 2005 and 2018 from 12 populations of *R. ferrumequinum*, and the sampling times of each population were similar. The samples included bat wing membranes or tissues from Beijing (BJ), Henan (HeN), Shanxi (HY, FD), Shandong (SD), Shanxi (SX), Zhejiang (ZJ), Jilin (JL1, JL2, JL3), Yunnan (YN), and Gansu (GS) provinces (Figure 1, Table S1). We used mist nets to capture bats in their habitats and used biopsy punches with a diameter of 3 mm to collect wing membrane tissues. After sampling, all bats were released. For those individuals that died unexpectedly, the muscle tissues were collected. All samples were stored in 95% ethanol solution at −80°C for later use. All field studies were approved by the National Animal Research Authority of Northeast Normal University, China (approval number: NENU‐20080416) and the Forestry Bureau of Jilin Province of China (approval number: [2006]178).

### 2.2 DNA amplification and sequencing

We extracted genomic DNA from all samples using a UNIQ‐10 column animal genomic DNA isolation kit (Sangon Biotech, Shanghai, China) following the manufacturer's standard protocol. Different bat species (GenBank accession numbers: LOC117020172, NW_017732037 and NW_017739577) were aligned using Geneious 9.0 to complement the conserved regions of intron 1 and intron 2. Then, species‐specific primers (0702‐1F: 5’‐GGGCGGATTAAAGATGAA‐3′) of intron 1 and intron 2 (0702‐1R: 5’‐ACACTGTGTCCCGAGCAT‐3′) were designed manually in Primer 5.0 (Lalitha, 2000) to amplify the whole exon 2 region by using the gene sequence of *R. ferrumequinum*. To confirm the accuracy of the designed primers, polymerase chain reaction (PCR) and gene cloning were used to amplify full‐length MHC class II‐DRB exon 2 in each bat from gDNA. More details are described in Li et al. (2021). We treated the sequence as a real allele when it was detected at least twice in independent clones, and it was then aligned to other bat species using Geneious.

Ultra‐deep sequencing technologies are essential for working with such highly complex gene families. Amplicons were obtained using the primers 0702‐1F and 0702‐1R, as well as the genomic DNA of *R. ferrumequinum*; the details are described in the supplemental information (Text S1). The amplicons were sequenced using an Illumina v2 kit (2× 300‐bp paired‐end reads) on an Illumina MiSeq platform by Shanghai Sangon Biotechnology Co., Ltd.

The paired‐end reads were merged using FLASH v1.2.11 (Magoč & Salzberg, 2011). Further raw read filtering was done using USEARCH (Edgar, 2010) by removing the reads with Phred quality scores below 20. Next, the AmpliSAS tool was used for the genotyping of MHC alleles. The AmpliSAS tool (Sebastian et al., 2016) was used for the final de‐multiplexing, clustering, and filtering of Illumina reads. Following the author's recommendations, we used a stepwise threshold clustering program (Stutz & Bolnick, 2014) implemented in the AmpliSAS web software (http://evobiolab.biol.amu.edu.pl/amplisat/index.php?amplisas) to distinguish real sequence variants from sequencing errors or PCR chimeras (Sebastian et al., 2016). We set the maximum number of alleles per amplicon to 10 because we used co‐amplifying primers. Variants of lengths exhibiting frameshifts relative to the expected length, noncoding variants, and chimeras were discarded. Default values were used for other parameters. The maximum number of reads per amplicon was set as 5000 due to the software restriction. After filtering by AmpliSAS, the obtained sequences were compared with available information in NCBI public datasets to ensure that the obtained sequences were MHC sequences of *R. ferrumequinum*. Then, MHC alleles were aligned in Geneious to check whether the stop codons existed and to remove intron regions from the alignments, retaining the full 270 bp length of MHC class II exon 2. All raw sequences were deposited into the NCBI SRA database (bioproject accession number PRJNA792677).

### 2.3 Datasets used in analyses

For comparison between MHC and neutral genetic variation, genotype data of seven microsatellite loci (Reffer 15, 17, 19, 22, 24, 27, and 28) were used from a previous study (Sun et al., 2013). The microsatellite data were collected from the same population consistent with the MHC data.

Considering the significant regional divergence in neutral markers in *R. ferrumequinum*, we analyzed the MHC variation at two levels, genetic lineages (NE, CE and SW) and populations (12 populations) (Vlček et al., 2016).

### 2.4 Recombination events

Historical recombination events were estimated using the RDP, GENECONV, BootScan, MaxChi, Chimaera, Siscan, and 3Seq algorithms as implemented in RPD4 (Martin et al., 2017). We identified recombination breakpoints based on the positive results of at least two different algorithms using a 100‐bp window size. The *p*‐value for a recombination event was set to 0.000005.

### 2.5 MHC‐DRB phylogenetic relationships

First, we assessed phylogenetic relationships among MHC alleles using a Bayesian inference approach implemented in MrBayes 3.2.7 (Ronquist & Huelsenbeck, 2003). The HLA‐DRB1 sequence (accession number: NG_029921) was set as the outgroup in the phylogenetic analyses. A total of 149 alleles were included in the analyses, with 59 identified alleles of *R. ferrumequinum* used in this study as well as published chiropteran DRB sequences selected at random, including 11 *Myotis* spp. alleles (*Myotis velifer* and *Myotis vivesi*) (Richman et al., 2010), 12 *Artibeus jamaicensis* alleles (Del Real‐Monroy et al., 2014), nine *Carollia perspicillata* alleles (Schad, Voigt, et al., 2012), 18 *Noctilio albiventris* alleles (Schad, Voigt, et al., 2012), seven *Hipposideros armiger* alleles (accession numbers: JF770403–JF770409), 19 *Rhinolophus episcopus* alleles, and 13 *Rhinolophus siamensis* alleles (Li et al., 2021). The JC + G model was selected as the best‐fitting model of nucleotide substitution using jModelTest 2 (Darriba et al., 2012) under the corrected Akaike information criterion. The program was run for 8 × 10^6^ generations with a sampling frequency of 100 and a 25% burn‐in.

Second, we constructed MHC haplotype networks using the statistical parsimony algorithm TCS implemented in PopArt 1.7 (Leigh & Bryant, 2015) to add a measurement of relationships among alleles.

### 2.6 Signatures of selection

To detect the presence of historical positive selection, two different methods that compare the rates of synonymous and nonsynonymous substitutions separately for single codons were used. First, we estimated the average synonymous (dS) and nonsynonymous (dN) substitutions per synonymous and nonsynonymous sites (Nei & Gojobori, 1986) using MEGA v.7.0 (Kumar et al., 2016). We used the Nei–Gojobori/Jukes‐Cantor method (Jukes & Cantor, 1969) with 5000 bootstrap replicates to calculate the overall average of dN/dS (ω) for all the sites, the putative antigen‐binding sites (ABS), and the non‐ABS. ABS and non‐ABS were identified by comparison with the human ABS of the HLA‐DRB1 (accession number: NG_029921) and chiropteran ABS of MHC‐DRB (Salmier et al., 2016). A Z‐test was used to detect the selection probability by comparing the null hypothesis of the selection parameter ω against strictly neutral (dN = dS).

Second, the analyses were performed using the HyPhy program implemented on the Datamonkey server (www.datamonkey.org) (Weaver et al., 2018). Alleles detected as recombination events in RDP4 were removed in subsequent analysis. Moreover, we checked for signatures of recombination using the genetic algorithm recombination detection method (Kosakovsky Pond et al., 2006) on the Datamonkey website before subsequent positive selection analysis, as recombination events can affect the outcome of selection tests. Two maximum‐likelihood methods were employed for detection of pervasive positive selection: fast, unconstrained Bayesian approximation (FUBAR) (Murrell et al., 2013), and fixed effects likelihood (FEL) (Kosakovsky Pond & Frost, 2005). In addition, a mixed effects model of evolution (MEME) (Murrell et al., 2012) that is capable of identifying instances of episodic positive or diversifying selection at the level of an individual site was also provided. The key difference between pervasive positive selection and episodic positive selection detected by the MEME model is that the former requires the mean dN/dS at a site to be >1 when averaged over time, while MEME also detects bursts of selection followed by conservation that often yield mean dN/dS < 1, which would be missed by conventional approaches (Murray et al., 2013). Significance levels of *p* < 0.1 in FEL, MEME, and a posterior probability of 0.9 in FUBAR were considered as indicating positively selected sites. We identified codons under pervasive positive selection based on the consensus of the FUBAR and FEL approaches.

### 2.7 Supertype clustering

We converted MHC alleles into supertypes by grouping functionally similar MHC alleles into the same supertypes based on their biochemical similarities at the amino acids that are known to interact with the antigen. Because the MEME approach was employed to detect different kinds of selection, we considered codons under positive selection based on the consensus of FUBAR and FEL approaches. Thus, 10 positive amino acid sites were substituted by a set of five physicochemical descriptors: z1 (hydrophobicity), z2 (steric bulk), z3 (polarity), z4, and z5 (electronic effects) (Sandberg et al., 1998). Alleles were clustered by discriminant analysis of principal components (DAPC) with the “adagenet” package in R 3.5.3 (Grunsky, 2002). This analysis implemented a k‐means clustering algorithm based on the Bayesian information criterion (BIC). The most probable number of supertypes in our data set was defined as the minimal number of clusters after which the BIC decreased by a negligible amount (Jombart et al., 2010). We retained all of the information, including 100 PCs, and K = 6 had the lowest BIC values (Figure S1).

### 2.8 Genetic diversity

For MHC data, six parameters were estimated for MHC polymorphism in each population. DnaSP v.6 (Rozas et al., 2017) was used to calculate the total number of unique alleles per population (h), the average nucleotide diversity (π), the number of segregating sites (S), and the number of private alleles (P). Moreover, the average number of alleles per individual (NaI) was calculated; this served as a simple proxy for individual heterozygosity, as described by Miller et al. (2010). We used these measures because we were unable to assign MHC alleles to specific loci when using co‐amplifying primers; thus, the heterozygosity could not be calculated. Therefore, all unique MHC sequence variants detected were treated as different alleles in this study, even though they may stem from different loci (Sagonas et al., 2019). Allele richness (A_R_) with a correction for sample size was estimated using a rarefaction method with the “hierfstat” package (Goudet, 2005) in R. MHC data used binary encoding as the presence/absence of an allele and created a 0/1 matrix with individuals in rows and MHC‐allele IDs in columns. Supertype diversity was estimated by the total number of supertypes carried by individuals at each sampling site.

For microsatellite data, we calculated microsatellite allelic richness (A_R_) using FSTAT 2.9.4 (Goudet, 2013) and both the observed and expected heterozygosity using GenAlEx 6.51 (Peakall & Smouse, 2012) for each population.

### 2.9 Comparison between MHC and microsatellite variation and genetic structuring

To assess the correlation between putative adaptive and neutral variation, we tested the correlations between MHC and microsatellite variation across the populations using the R package “psych” (Spearman's rank correlation was used due to some parameters not conforming to a normal distribution), and then used the p.adjust function of the “fdrci” R package to adjust the *p* values (Millstein et al., 2022).

To investigate whether spatial variation in selection has influenced the geographical variation of MHC class II DRB genes, we compared the genetic structures between MHC and microsatellites across populations. First, for both markers, population differentiation was calculated using Jost's D (Dest) (Jost et al., 2018) by SpadeR (Chao et al., 2016), a method that is suitable for genotype data with multiple alleles per locus in each individual to measure the degree of genetic differentiation. Jost's D was calculated based on allele frequencies, and the allele frequency of the MHC gene was calculated by Arlequin 3.5 (Excoffier & Lischer, 2010) based on the allele sequences (Loiseau et al., 2009; Miller et al., 2010; Pearson et al., 2018; Strand et al., 2012). Direct comparisons of Dest values are not reliable due to the different evolutionary processes of MHC and microsatellites. Thus, a bootstrap analysis was used to estimate the standard error of all statistics and produce their confidence intervals (Kim & Kim, 2017), a method that has been used previously in the comparison of genetic differentiation between MHC and microsatellites (Agudo et al., 2011; Li et al., 2016; Loiseau et al., 2009; Trujillo et al., 2021). We used 5000 bootstrap replicates of all pairwise population Dest values for the MHC and microsatellite data sets and evaluated the overlap between the means of the bootstrapped distributions, and a histogram was used to represent the distribution of these means. The details and code are described by Trujillo et al. (2021). Subsequently, the 95% confidence intervals were calculated by bootstrap replicates to estimate the statistical significance between MHC and microsatellite genetic differentiation. In addition, we compared the all pairwise population Dest values between MHC and microsatellites by estimating the microsatellite‐based Dest values with 95% confidence intervals (CI) using the “rmisc” R package. Then, each MHC‐based pairwise Dest value was compared with the 95% CI of the microsatellites at both population and genetic lineage levels (Agudo et al., 2011; Loiseau et al., 2009; Weir, 1996). Moreover, we used non‐metric multidimensional scaling (NMDS) performed in PAST (Hammer et al., 2001) to visualize and compare matrices of pairwise Dest values based on MHC and microsatellites.

Second, to assess patterns of isolation by distance (IBD), we performed Mantel tests to estimate the relationships between Dest/(1 − Dest) and geographic distances for MHC and microsatellites independently. We also tested whether the slopes of the regression between Dest/(1 − Dest) and geographical distance based on the two markers were significantly different by using the “simba” package (Jurasinski & Retzer, 2012) in R. In addition, a partial Mantel test was used to compared Dest/(1 − Dest) between MHC and geographic distances while controlling the neutral genetic distance (Dest/(1 − Dest)). Mantel tests and partial Mantel (Spearman statistics) tests were performed with 9999 permutations using the “vegan” package (Dixon, 2003) in R. Geographic distances between populations were determined from GPS coordinates recorded at each collecting site and were converted into a matrix by Geographic Distance Matrix Generator v1.2.3 (Ersts, 2017).

Finally, we tested the population structure displayed at the MHC‐DRB locus and microsatellites. To further show differences within and between three genetic lineages as far as possible while minimizing variation within clusters, DAPC was implemented in the R package “adegenet” for MHC and microsatellites independently. BIC was used to assess the optimal number of clusters for DAPC analysis. The number of principal components (PCs) retained in DAPC was chosen to maximize the α‐score (using the optim.a.score function of adegenet). According to the α‐score optimization, 35 PCs and 11 discriminant functions were retained for MHC, and 11 PCs and 2 discriminant functions were retained for microsatellites.

### 2.10 Effects of climatic factors on MHC polymorphism and genetic distance

Climatic parameters were considered to reflect pathogenic landscape variation to an extent. To determine the relative contributions of geographical distance and climatic factors to the genetic differentiation and MHC variants, we first downloaded 19 bioclimatic variables (refer to Table S2 for details) for each population from CHELSA (resolution: 30 arcsec, ~1 km; time period: 1979–2013) (Karger et al., 2017) using the R package “raster” v.2.8–4 (Hijams, 2017). The bioclimatic variables included temperature and precipitation (Table S2).

Second, considering the multicollinearity problems in MHC variation and bioclimatic variables, we used variable clustering to assess the redundancy of the environmental variables by the “varclus” procedure in the “hmisc” R package. We considered a Spearman's rho (ρ^2^) of <0.5 as indicating that no multicollinearity existed. Thus, the parameters of MHC variations (π, NaI, P, and S) and the number of supertypes (ST2, ST4, ST5, and ST6) were retained for the following analysis with ρ^2^ values <0.5 (Figure S2). For bioclimatic variables, according to the results of Spearman correlation, we retained BIO2 (monthly mean temperature), BIO3 (isothermality), BIO8 (mean temperature of wettest quarter), and BIO15 (precipitation seasonality) from the 19 climate factors for the next step in the analysis (Figure S2). All factors were first standardized by the MuMIn package function “stdize” so that the subsequent regression coefficients of all independent variables could be placed at comparable levels to assess their relative importance.

Third, we incorporated the climate factors, geographic distance, and MHC variation into the multiple regression distance matrices (MRM) (Lichstein, 2007) as implemented in the R package “ecodist” (Goslee & Urban, 2007). MRM is a derivative of the Mantel test that allows for multiple variables within the model. Moreover, we also tested correlations between MHC genetic differentiation and climate factors, as well as geographic distance, using MRM.

Fourth, we used multinomial log‐linear (MNL) models to further verify the influence of each climate factor on different MHC variant parameters in the R package “nnet,” and analysis of variance was used to obtain the *p*‐value of the model. We ran the model with the climate variables and MHC variants. Each MHC variant was considered in the MNL model as an independent response variable and climate variables as independent variables. The model syntax was as follows: Multinom (MHC variants~BIO2 + BIO3 + BIO8 + BIO15).

Finally, considering that phylogeographic groups of *R. ferrumequinum* may be correlated with climate variables, we conducted two analyses. (1) The partial Mantel test (Spearman statistics) was used to estimate the correlation between climate factors and MHC diversity with 9999 permutations using the “vegan” package (Dixon, 2003) in R, while controlling for the genetic structure of microsatellites. The genetic structure of microsatellites was assessed by STRUCTURE v.2.3.4 (Pritchard et al., 2000) using 5000 iterations after 10,000 burn‐in iterations. The maximum K value was set to the number of populations, with five runs for each K value. STRUCTURE HARVESTER v.0.6.94 (Earl, 2012) was used to determine the most likely number of clusters using the delta K method. (2) Phylogenetic generalized linear model (PGLS) regression (Symonds & Blomberg, 2014) was used to investigate the relationship between the climate factors and MHC diversity while statistically controlling for phylogeny. PGLS regression analyses were performed using R with the “ape” packages. PGLS is a model in which the covariance (correlation) structure between species is permitted to match that expected under a Brownian motion process of evolution on the tree. The simplest generalization of the Brownian model is one with one additional parameter to scale the expected covariance under pairs of species. This model is called the λ model of Pagel, or Pagel's λ. The value of λ usually ranges from 0 to 1, where 0 is equivalent to no phylogenetic structure, and 1 is equal to the actual phylogenetic structure. Because neutral markers are more suitable for assessing phylogeographical structure, the phylogenetic tree of *R. ferrumequinum* was constructed using microsatellite data with the Neighbor‐Joining method in Population v12.30.

## 3 RESULTS

### 3.1 MHC‐DRB locus amplification and genotyping

In total, we obtained 19,824–165,756 MiSeq sequence reads per individual in the raw data and 19,733–164,547 sequence reads after quality control (Table S1). After genotyping, full‐length sequences of 270 bp of MHC II‐DRB exon 2 were obtained from 121 bats, and 59 alleles were identified (Rhfe 01–59, GenBank accession number: OM066672‐OM066730). Surprisingly, we found indels in a large number of alleles. A total of 31 alleles had 3‐bp insertions in 249–252 bp, and 6 alleles had 3 bp absent in 218–221 bp (Figure S3). Such indels of single codons did not result in shift mutations or codetermination codons, and none of the alleles showed the signals of pseudogenization. Therefore, all alleles were considered as functional alleles in downstream analyses.

### 3.2 MHC diversity in populations and lineages of *R. ferrumequinum*

The maximum number of MHC class II alleles per individual was seven (*n* = 1–7), indicating the presence of at least four class II loci in *R. ferrumequinum*. The number of MHC alleles (h) varied substantially across populations, from 6 in ZJ to 68 in FD and JL2. The NaI of *R. ferrumequinum* ranged from 0.17 to 0.565 per population. However, other MHC polymorphism parameters showed little difference between populations (Figure 1, Table S5). No allele was common to all populations, and the populations carried from 0 to 8 private alleles, with YN having the highest number of private alleles. As for the lineages of *R. ferrumequinum*, the AR of SW lineage had the highest value (AR_SW_ = 1.717) in all lineages, and SW also had the highest number of private alleles, but the NaI value was the lowest. The NE lineage tended to have the lowest numbers of private alleles and AR values, and the CE lineage showed the highest number of private alleles and AR, NaI values (Figure 1, Table S4).

All of the 59 MHC alleles were clustered into six supertypes, with each supertype grouping 2 to 17 of the original alleles. The number of supertypes per population was within a narrow range from three to six. BJ, HeN, and YN had all of the supertypes, while JL2, JL3, and ZJ only had three supertypes, and ST1, ST3 and ST4 were present in all populations. For the lineages of *R. ferrumequinum*, CE and SW had all of the supertypes, and NE lineages had four supertypes, different numbers of MHC supertypes were exhibited among the lineages (Figure 1).

### 3.3 MHC‐DRB recombination events and historical selection

Recombination events were detected on Rhfe 19 and 49 by RDP. These two haplotypes were eliminated in subsequent positive selection analysis, and no recombination events were detected in the rest of the alleles by genetic algorithm recombination detection analyses.

The FEL and FUBAR models evaluated pervasive selection sites over the entire evolutionary history of the population and showed similar results. In *R. ferrumequinum*, 10 positively selected sites were consistent between the two models (Figure S4); 11/15 positively selected sites of FUBAR were consistent with the chiropteran and human ABS, as were 8/11 positively selected sites of FEL (Figure S4). The episodic positive selection that was measured by MEME showed that 9/19 positively selected sites were consistent with pervasive selection sites detected by the FEL and FUBAR models. In addition, 11/19 episodic positive selected sites were consistent with the chiropteran and human ABS (Figure S4).

The ratios of dN and dS were significantly different from neutrality in the ABS (dN/dS range: 2.243–6.972; *p* < 0.05 for each population) and non‐ABS (dN/dS range: 1.06–3.669) in all populations, but no significant difference was found in BJ or ZJ. In total, significant positive selection was detected at the ABS of *R. ferrumequinum* (Z = 5.22, *p* = 0.001), and no significant negative selection (Z = 0.35) was detected at the non‐ABS (Table S3).

### 3.4 MHC phylogeny

**FIGURE 2:**
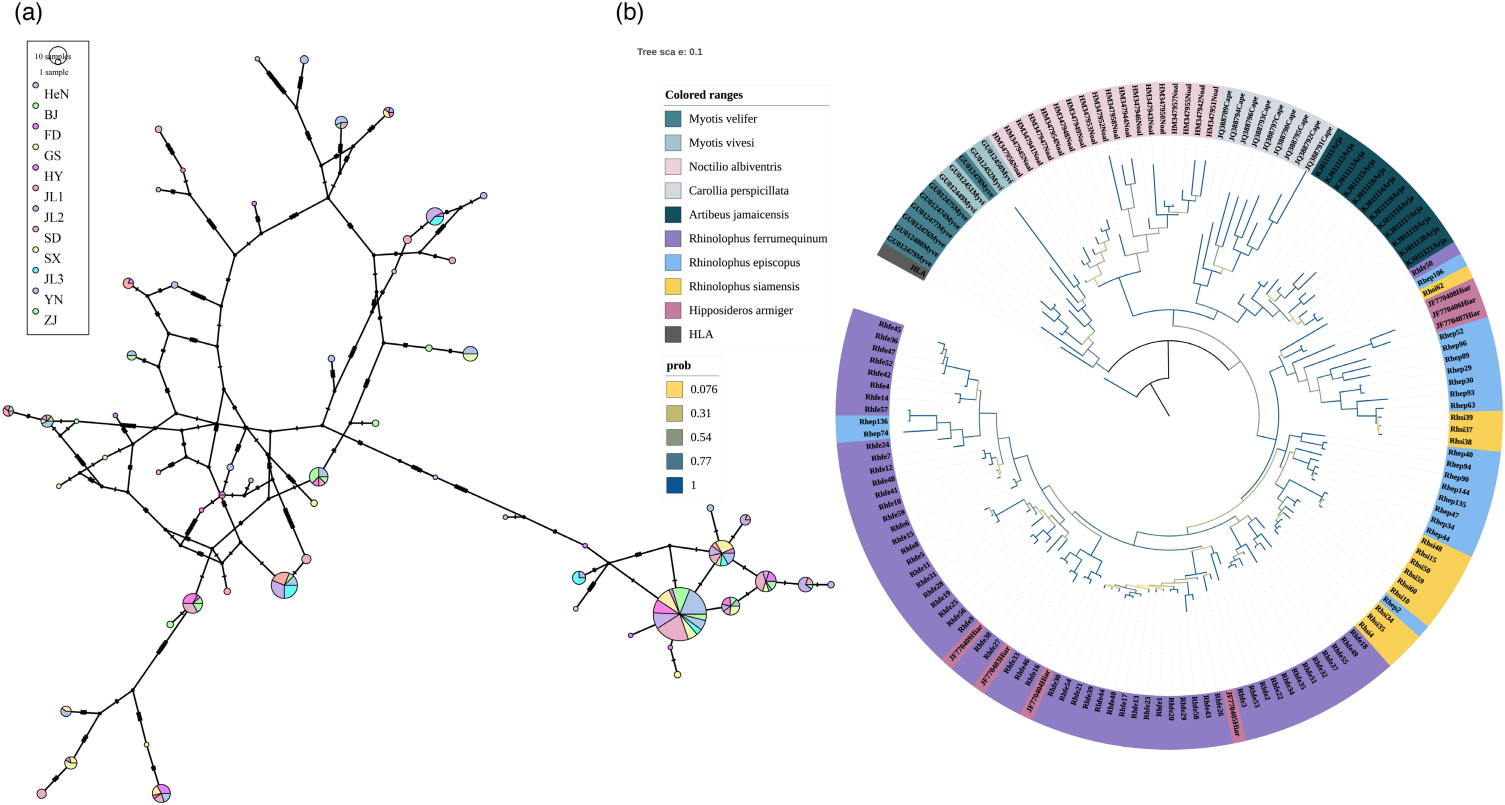
(a) Haplotype networks of MHC II ‐DRB exon 2 alleles at the nucleotide level from *R. ferrumequinum*. Nodes are proportional to the number of bats carrying each haplotype and are colored by the environments where the bats were trapped (see legend). Hatch marks represent mutations. Interruptions in lines indicate the presence of more than ten mutations. (b) Phylogenetic relationships of MHC II‐DRB exon 2 alleles from *R. ferrumequinum* and other chiropterans based on a Bayesian approach.

The network showed that one haplotype named Rhfe01 was absent in all the bats except those of the JA population, and a large number of haplotypes were found among the populations, suggesting that the MHC alleles may have generated new genes during the ancient gene duplication events and that these subsequently evolved independently. A phylogenetic tree was constructed from MHC II‐*DRB* exon 2 alleles of nine bat species; the resulting tree exhibited that *R. ferrumequinum* was intermixed with *H. armiger*, *R. episcopus*, and *R. siamensis* (Figure 2) and showed trans‐species evolution.

### 3.5 Comparison between MHC and microsatellite variation and genetic structuring

Spearman's correlation analysis revealed no significant correlations between the diversity parameters of MHC and microsatellites.

**FIGURE 3:**
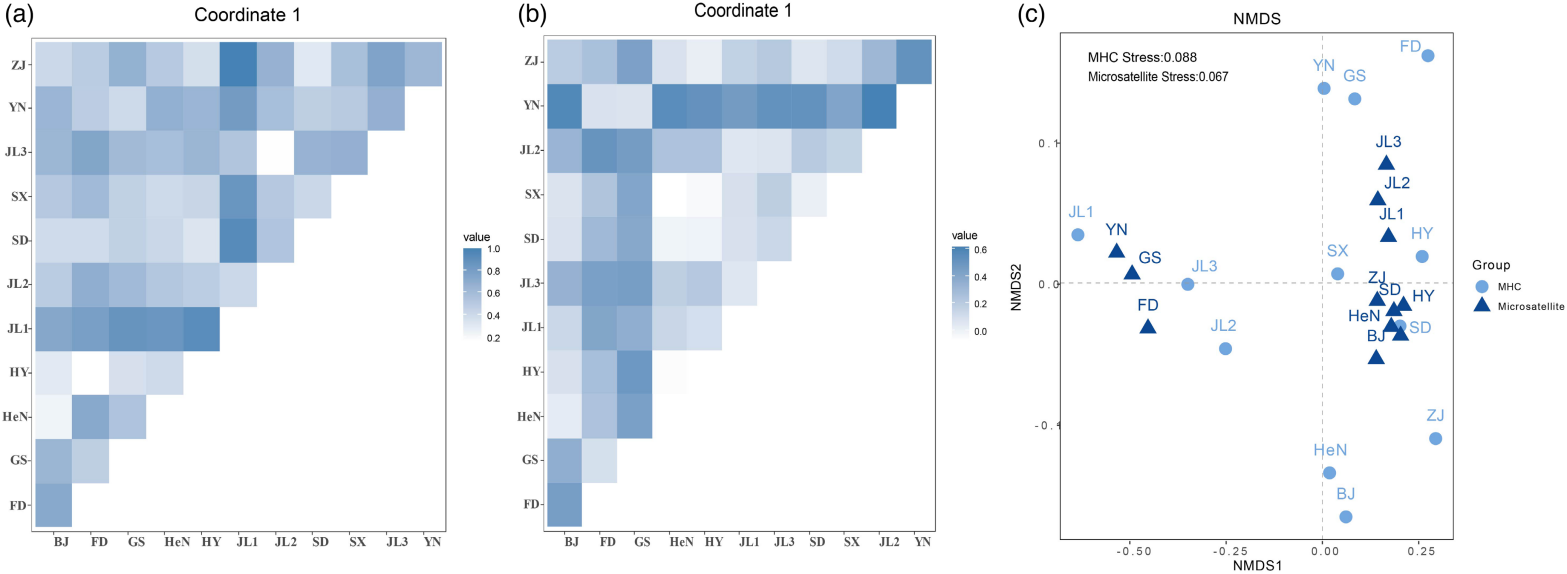
Pairwise population differentiation between 12 populations based on the Dest values (a) MHC II‐*DRB* exon 2; (b) microsatellite; (c) non‐metric two‐dimensional scaling of the *R. ferrumequinum* pairwise Dest values matrix.

**FIGURE 4:**
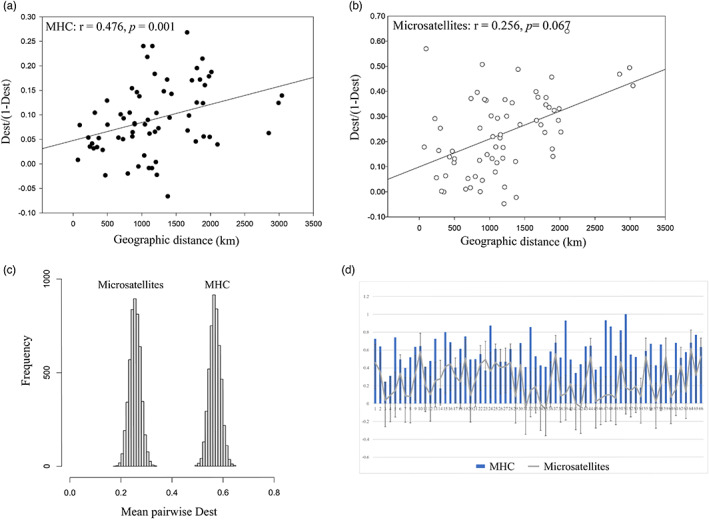
(a) Correlations between MHC pairwise Dest/(1− Dest) values and geographic distances (km). (b) Correlations between microsatellite pairwise Dest/(1− Dest) values and geographic distances (km). (c) The distribution of 5000 bootstrapped replicates of mean pairwise Dest values derived from microsatellites (left) and the MHC locus (right). (d) Comparisons of Dest values of microsatellites to MHC class IIB for each pairwise population in this study. Error bars indicate 95% confidence intervals and were estimated using bootstrapping.

The genetic differentiation between populations for MHC loci varied substantially, with Dest values from 0.17 (FD vs HY) to 1 (JL1 vs ZJ) (Figure 3a,b). NMDS also exhibited lineage differentiation, where populations within the same lineage were closely spaced (Figure 3c). In contrast, pairwise Dest values for the microsatellite loci ranged from 0 to 0.618, which was lower than the Dest values of MHC. Bootstrap analysis yielded mean Dest values of MHC that were consistently larger than those from microsatellites (Figure 4c), and the 95% confidence intervals were from −0.162 to −0.465, a range that ruled out a difference of zero. Because the 95% (0.95) confidence interval for that difference failed to contain zero, the difference between MHC and microsatellite genetic difference was statistically significant. Moreover, 47 pairwise Dest values of microsatellites (Microsatellite 95% CI: 0.206–0.295) from 66 population pairs were above the upper limits of those of MHC (Figure 4d), indicating the diversifying selection. However, some paired population comparisons showed the opposite pattern (Figure 4d), suggesting balancing selection as well as some homogenizing directional selection.

The Mantel test showed a significant correlation between Dest values of MHC and microsatellites (*r* = 0.375, *p* = 0.017). The pattern of isolation by distance was significant for MHC (*r* = 0.476, *p* = 0.001, Figure 4a), but not for the microsatellites (*r* = 0.256, *p* = 0.067, Figure 4b). The IBD regression slope of MHC was significantly steeper than that of microsatellite (slop_MHC_ = 0.0008, slop_microsatellites_ = 0.0003, *p* = 0.001). The partial Mantel test showed that there was still a strong relationship between pairwise Dest (MHC) and geographic pairwise distances, even after controlling for the effects of neutral microsatellites (*r* = 0.338, *p* = 0.028).

For genetic lineages of *R. ferrumequinum*, Dest values of MHC showed medium differentiation between NE and the other two lineages (Dest_MHC_: CE vs NE = 0.482; NE vs SW = 0.495) and relatively low differentiation between CE and SW (Dest = 0.232). Genetic differentiation of microsatellites showed different patterns, with medium differentiation between CE, NE, and SW lineages (Dest_microsatellites_: CE vs SW = 0.409; NE vs SW = 0.443) and low differentiation between CE and NE (Dest_microsatellites_ = 0.167). In both markers, medium differentiation occurred between NE and SW. However, the genetic differentiation was higher between CE and NE for MHC than for microsatellites, and the level of genetic differentiation was lower between CE and SW for MHC than for microsatellites. Although the MHC genetic differentiation was greater than in microsatellites, pairwise Dest values of MHC fell within the 95% confidence interval of microsatellites (microsatellite 95% CI: 0–0.714), and no significant difference was shown by the bootstrap method for either of the markers.

The results of DAPC based on MHC showed an admixture structure between populations but three distinct clusters of genetic lineages. For microsatellite loci, the results of DAPC showed no clear structural characteristics between populations but separated the SW lineage along the first (horizontal) axis and showed partial overlapping between NE and CE lineages (Figure S5). In contrast with the DAPC results for MHC, we observed a similar situation between the two genetic markers in that both had separate clear structures in the lineages of *R. ferrumequinum*.

### 3.6 Effects of climate variables on MHC diversity and genetic differentiation

In both univariate and multivariate MRM analyses, we found significant correlations between MHC variants and climate variables (Table 1). MNL analysis further supported the results of MRM, and there was a clear positive correlation of the univariate climate factors (BIO2, BIO3, and BIO15) on the number of supertype and MHC private alleles in *R. ferrumequinum* from the current study range. Significant relationships were detected between P and three climate factors (BIO2 [monthly mean temperature], BIO3 [isothermality], and BIO15 [precipitation seasonality]), ST2 and BIO15, ST5, and BIO2, as well as ST6 and BIO3 (Table 2).

**TABLE 1:** Results of multiple linear regression models (MRM) between MHC diversity or MHC genetic distance and environmental distance

	MHC diversity					MHC genetic distance			
	*R* ^2^	*F*	Coef	*p* value		*R* ^2^	*F*	Coef	*p* value
Univariate MRM					Univariate MRM				
Climate	0.302	27.72	0.857	**0.005**	Climate	0.015	0.985	0.159	0.419
SDM	0.001	0.075	−0.185	0.809	SDM	0.002	0.136	0.085	0.734
Multivariate MRM (~climate+SDM)					Multivariate MRM (~climate+SDM)				
Climate	0.309	14.075	0.884	**0.005**	Climate	0.016	0.500	0.154	0.457
SDM			−0.185	0.932	SDM			0.039	0.875

*Note*: Boldface indicates *p* values that are significant after Bonferroni–Holm correction (P_B‐H_ < 0.05). Climate, climate factors matrix based on CHELSA after varclus analysis; SDM, spatial distance matrix based on PCNMs.

**TABLE 2:** Results of a multinomial logit model (MNL) describing the effects of climate factors on MHC polymorphism (P and ST)

Response	P			ST6			ST2			ST5		
	LR Chisq	*p* value	AIC	LR Chisq	*p* value	AIC	LR Chisq	*p* value	AIC	LR Chisq	*p* value	AIC
BIO2	17.700	**0.007**	60.000	4.683	0.321	40.000	0	0.999	20.000	12.471	**0.014**	48.318
BIO3	25.089	**0.00033**		12.715	**0.013**		2.632	0.268		5.280	0.260	
BIO8	5.546	0.475		0.0001	1		0.006	0.007		5.054	0.282	
BIO15	15.186	**0.019**		0.0004	1		6.444	**0.040**		4.894	0.298	

*Note*: Boldface values indicate *p* < 0.05. MHC polymorphism abbreviations are the same as in Figure 1.

Genetic differentiation of MHC was not correlated with any climate factor or geographic distance in the MRM analysis. Moreover, the partial Mantel test showed a significant correlation (*r* = 0.439, *p* = 0.008) between MHC diversity and climate factors when controlling for the genetic structure of microsatellites.

Similar to the results using the MNL model, The PGLS regression showed that three climate factors (BIO2, BIO3, and BIO15) affected ST2, ST5, ST6, and P. In addition, the effect of climate factors on ST2 and ST5 was not influenced by phylogenetic signal (λ around 0) (Table 3). However, the effects of climate factors on ST6 and P were influenced by a strong phylogenetic signal (λ > 1). The value of the regression coefficients was close to 0.05, and the *p*‐value was close to 0.01, indicating a linear relationship between variables and that the null hypothesis could be rejected, meaning that the variables were not affected by the phylogenetic relationship (Table 3).

**TABLE 3:** Results of phylogenetic generalized least squares (PGLS) describing the effects of climate factors on MHC polymorphism and controlling for the influence of phylogeny (P and ST)

Response		BIO2	BIO3	BIO8	BIO15
P	Value	−0.090	0.048	0.058	0.011
	*p*‐value	0.119	**0.0001**	**0.009**	0.282
	lambda	1.2			
	AIC	69.102			
ST6	Coef	0.017	0.019	0.057	0.016
	*p* value	0.719	**0.021**	**0.017**	0.376
	lambda	0.976			
	AIC	68.821			
ST2	Coef	0.003	0.011	0.013	0.025
	*p* value	0.850	**0.021**	0.292	**0.069**
	lambda	−0.297			
	AIC	59.017			
ST5	Coef	0.108	0.001	0.005	−0.025
	*p* value	**0.016**	0.899	0.855	**0.077**
	lambda	−1.924			
	AIC	68.871			

*Note*: Boldface values indicate *p* < 0.05. MHC polymorphism abbreviations are the same as in Figure 1. Lambda indicates the degree of phylogenetic influence.

## 4 DISCUSSION

### 4.1 Characteristics of MHC alleles

Gene copy number variation in the MHC is thought to be important in adaptation to different levels of parasite diversity (Meyer‐Lucht et al., 2016) and has been shown to compensate for low allelic variation (Meyer‐Lucht et al., 2016; Otting et al., 2005; Siddle et al., 2010). For instance, Siddle et al. (2010) found that overall levels of MHC allelic diversity were low in Tasmanian devils, although copy number differences provided present evidence of variation in MHC rather than sequence polymorphism. The number range of MHC alleles in this study was from one to seven variants per individual, suggesting gene copy number variation in the *R. ferrumequinum* MHC‐DRB locus, meaning that at least four MHC‐DRB loci were detected. Copy number variation has been observed in the MHC genes of almost all vertebrate groups (Alcaide et al., 2014; Eimes et al., 2011; Jaratlerdsiri et al., 2014; Lighten et al., 2014; Schad, Voigt, et al., 2012; Siddle et al., 2010). Compared with other bat species, except for 10 loci in the sac‐winged bat (Schad, Voigt, et al., 2012), 17 loci of *R. episcopus*, and seven loci of *R. siamensis* (Li et al., 2021), 1 to 3 loci have been found in most studied bat species (Del Real‐Monroy et al., 2014; Richman et al., 2010; Salmier et al., 2016; Schad, Voigt, et al., 2012; Yi et al., 2020). However, it is worth noting that we genotyped MHC alleles using AmpliSAS. Due to computational limitations, the maximum amplicon depth set of AmpliSAS is 5000 reads, which may have slightly underestimated the number of alleles (Rekdal et al., 2018).

A large number of indels were found in the sequences of *R. ferrumequinum*. This may suggest a common characteristic or an evolutionarily important trait of MHC in bats (Qurkhuli et al., 2019), as a similar situation has been found in other *Rhinolophus* bats (Li et al., 2021) and other chiropteran bats such as *C. perspicillata* and *D. rotundus* (Salmier et al., 2016).

Functional MHC supertypes have been demonstrated to be associated with different types of particular pathogens and host susceptibility, and thus supertypes are valuable indicators of pathogen‐mediated selection. In this study, the clustering of alleles into six MHC supertypes by functional properties with signals of selection was detected. Supertypes showed different proportions among populations and genetic lineages, implying local adaptation. Moreover, the NE lineage only owned four MHC supertypes and had the lowest number of private MHC alleles, possibly due to contrasting pathogen pressures in these sites (Phillips et al., 2018; Savage & Zamudio, 2011, 2016; Sepil et al., 2013; Smallbone et al., 2021; Wang et al., 2017). *Rhinolophus ferrumequinum* is subject to pathogen pressure from *Pseudogymnoascus destructans*, the causative agent of white‐nose syndrome, during the hibernation period (Hoyt et al., 2016; Li et al., 2022). The populations from the NE and CE lineages have a longer hibernating time than SW, suggesting that NE and CE might have stronger pathogen pressure from *P. destructans* than SW, even if this bat species has high resistance to the infection (Hoyt et al., 2016; Li et al., 2022). This case could lead to a large number of alleles being shared between NE and CE lineages. Besides the pathogen stress of *P. destructans*, *Toxoplasma gondii* has also been reported to infect *R. ferrumequinum*, and the prevalence of *T. gondii* was higher in bats in southern China (Guangdong and Jiangxi) than in northern China (Jilin and Liaoning) (Qin et al., 2014). A possible reason for this is that the warm and humid environment in southern China is more suitable for the survival of *T. gondii* oocysts (Dubey, 2010; Qin et al., 2014). The SW lineage is isolated from the CE lineage by the Qinling Mountains (Sun et al., 2013). Limited gene flow and heterogeneity in pathogen pressure may lead to the local adaptation of populations to different pathogens and facilitate diversifying selection operating on MHC geographical variation (Ekblom et al., 2007; Herdegen et al., 2014; Li et al., 2016; Loiseau et al., 2009; Miller et al., 2010). Among different populations of *R. ferrumequinum*, as well as in genetic lineages, no consistent genetic pattern was found, suggesting that *R. ferrumequinum* has experienced local adaptation at the microevolutionary level at different geographical scales.

### 4.2 Historical positive selection

Long‐term historical selection operating in the past was evident from the trans‐species polymorphism signals and positive selection signals obtained from the site‐specific test results. First, we found that MHC alleles were intermixed between different bat species and *R. ferrumequinum* (Figure 2), suggesting their trans‐species evolution (TSP). Although TSP may be subjected to the neutral possibility of incomplete lineage sorting over a short period of time, TSP selected for long‐term historical balancing selection can be maintained the same advantageous MHC alleles for millions, or even tens of millions, of years (Klein et al., 1998; Těšický & Vinkler, 2015). In this study, the bat species have been diverged more than million years (Flanders et al., 2011; Zhang et al., 2018), suggesting TSP could be maintained by long‐term historical balancing selection of MHC genes. Strong evidence for TSP in MHC IIB genes has been found in a wide range of taxa, particularly those of mammals and fish (Garrigan & Hedrick, 2003; Graser et al., 1996; Klein et al., 1993; Lenz et al., 2013). Multiple MHC loci were co‐amplified in this study; thus, distinguishing between trans‐species polymorphism and gene duplication may be impossible. Second, we found evidence of a high dN/dS ratio, especially in sites involved in antigen binding (Table 3). Previous studies have suggested that selection acting on ABSs that is responsible for antigen recognition (Piertney & Oliver, 2006) may be primarily from foreign pathogens and indicates historical positive selection in polymorphic MHC genes (Hughes & Yeager, 1998). The presence of these historical selection signatures does not mean that selection is acting contemporarily on the MHC genes but does indicate that selection played an important role over an evolutionary time scale.

### 4.3 Evolutionary forces driving the spatial pattern of MHC variation

The difficulty of disentangling selection in MHC is the mixture of multiple selective forces that are not mutually exclusive and that can operate simultaneously at different geographic and evolutionary scales. One way to test for selection is to compare the genetic structure of the MHC with that of neutral loci in multiple populations. Balancing selection is usually thought to maintain high levels of MHC diversity, resulting in a lower genetic structure of MHC in populations than in neutral loci (Piertney & Oliver, 2006; Sommer, 2005; Spurgin & Richardson, 2010). If adaptations to local pathogenic fauna result in diverse MHC allele pools being maintained in populations or species, these genes will display a more pronounced population structure than neutral genes, and this could indicate diversifying selection (Herdegen et al., 2014; Spurgin & Richardson, 2010).

By comparing the neutral and adaptive markers of *R. ferrumequinum*, we found that diversifying selection affected the MHC genes to a greater extent than demographic processes. The extent of the population subdivision in MHC loci exceeded that observed at microsatellite loci among both populations and genetic lineages, indicating diversifying selection. However, there were significant correlations in genetic differentiation between two markers indicating the contribution of selection to MHC variation, since if the MHC behaved neutrally, the Dest values of MHC would be positively correlated with neutral microsatellites. However, the partial Mantel test was performed where pairwise Dest values at MHC loci were correlated with geographical distance while keeping constant differentiation at microsatellites. This test should provide evidence in favor of a significant positive correlation between geographical distance and MHC differentiation that is independent of demographic and stochastic factors, indicated a role of selection (Ekblom et al., 2007; Inga et al., 2014; Kyle et al., 2014; Loiseau et al., 2009; Matthews et al., 2010; Niskanen et al., 2014). Moreover, the NMDS and DAPC results for both markers revealed that populations were generally clustered by geographical area, and a clear structuring was found by clustering the lineages of *R. ferrumequinum*. This situation further supports our interpretation that the regionally differentiated populations we observed could also be interpreted as evidence for adaptation to local environments and local parasite assemblages, implying diversifying selection, which have been described in several other species (Ekblom et al., 2007; Kyle et al., 2014; Li et al., 2016; Loiseau et al., 2009; Matthews et al., 2010; Sagonas et al., 2019).

However, comparing the MHC‐based Dest and microsatellite‐based Dest values within the 95% CIs within genetic lineages and some populations, no significant differences were found between the two markers, suggesting the effects of balancing selection and some homogenizing directional selection (Agudo et al., 2011; Li et al., 2016; Loiseau et al., 2009). Most previous studies have obtained inconsistent results across different populations of the same species. Especially in wild populations, species are subjected to different selection effects at different geographical scales, for example, salmon, guppies, and amphibians (Herdegen et al., 2014; Landry & Bernatchez, 2001; Miller et al., 2001; Talarico et al., 2019). The authors ascribed these differences to the dominant role of diversifying/fluctuating selection at the smaller scale and an interplay between (weak) drift and balancing selection at the larger scale.

### 4.4 Effects of climatic factors on the adaptive evolution in MHC genes

The phenotypic characteristics of *R. ferrumequinum* and other bat species have been shown to co‐evolve with the ecological environment (Jacobs et al., 2017; Jiang et al., 2015; Mutumi et al., 2016; Sun et al., 2013), indicating the selection pressure from the environment leading to adaptive evolution. Our results also demonstrated that MHC variation could be affected by climate variables, and thus the analysis has yielded further supporting evidence to explain the adaptive evolutionary processes of *R. ferrumequinum*.

In this study, we found a significant relationship between MHC polymorphism and climatic factors, including those representing temperature and humidity, in both MRM and MNL analyses. The MNL results further demonstrated that climate factors could affect the number of MHC private alleles and supertypes. Notably, after excluding the influence of phylogenetic structure and phylogeny of *R. ferrumequinum* by the partial Mantel test and PGLS analysis, MHC polymorphism was still affected by climatic factors, suggesting the effect of local adaptation. Moreover, several specific climatic factors influenced the MHC supertypes. For example, ST6 and ST5 could be positively affected by temperature, while ST6 was positively affected by precipitation. Given that supertypes are generally considered as valuable indicators of the host's susceptibility relationships to particular pathogens, the association between a particular supertype and temperature or precipitation may be due to changes in climatic conditions that affect pathogen abundance or intensity, and thus pathogen and host interactions (Awadi et al., 2018; Brunner & Eizaguirre, 2016; Stefanović et al., 2021). In this study, the habitat characteristics of the bats varied greatly, and the interactions between environmental conditions were complex. These differences may lead to completely different selection pressures on populations or genetic lineages. For instance, *R. ferrumequinum* from NE will hibernate in the face of low temperature in winter. In contrast, some CE and SW populations experience a mild climate in winter and do not face prolonged low temperatures. However, bats may be disturbed by pathogens during hibernation. In existing studies, it has been found that during hibernation, *R. ferrumequinum* can be infected by *P. destructans* (Hoyt et al., 2016, 2020; Li et al., 2022), and some immunity‐related genes such as IL1B, H2‐Q10 and LYZ were under positive selection (Zhao et al., 2019). Therefore, we inferred that *R. ferrumequinum* may be strongly influenced by the ecological environment.

Moreover, a previous study has already found significant effects of mean annual temperature and precipitation on the distribution of some MHC proteins/alleles in hares (*Lepus capensis*), suggesting regional and climate effects (Awadi et al., 2018). In Atlantic salmon (Dionne et al., 2007), researchers found that allelic diversity of MHC increased with temperature. In research on golden jackals (*Canis aureus*), a clear positive effect of the temperature factor on the presence of MHC genotypes was found, and the MHC heterozygous individuals were more often found at locations with lower temperatures during the wettest period of the year (Stefanović et al., 2021). Therefore, we speculated that the present observed effect of climate variables on MHC variation may be interpreted as a signal of local adaptation to supposedly geographically varying pathogen and parasite selection pressures.

In conclusion, we investigated the adaptive MHC gene of *R. ferrumequinum*, with widespread geographical distribution and three genetic lineages. Through comparing an adaptive genetic marker with a neutral marker, we found that diversifying selection has had a major effect on MHC genes rather than demographic processes among populations, and some homogenization selection may have influenced the genetic lineages of *R. ferrumequinum*. By integrating climate factors, genetic structure, and MHC variation and supertypes, it appears that ambient temperature and humidity may act as putative fitness factors affecting MHC variants. This will increase our understanding of the importance of adaptive genetic variation in wild animal populations and the relationship with climate factors, and the supertypes analysis also provides new ideas for animal conservation. The results may help to conserve wild populations to cope with climate change or improve the disease resistance of wild animals through genetic manipulation technology (Johnson et al., 2016).

## CONFLICT OF INTEREST

We declare that we do not have any commercial or associative interest that represents a conflict of interest in connection with the work submitted.

## Supporting information


Appendix S1.

## Data Availability

All raw sequences were deposited into the NCBI SRA database (bioproject accession number PRJNA792677).
