# Supplementary material for: Diversifying selection and climatic effects on major histocompatibility complex class II gene diversity in the greater horseshoe bat
Source: Evol Appl. 2023 Jan 8;16(3):688–704. doi: 10.1111/eva.13528 (PMC10033860; doi:10.1111/eva.13528)
Supplement: Supplementary file 1 — Appendix S1. [file EVA-16-688-s001.docx]

**Diversifying selection and climatic effects on major histocompatibility complex class II gene diversity in the greater horseshoe bat**

**Supporting Information**

**TABLE S1** Sampling information of *R. ferrumequinum* used in this study.

**TABLE S2** The codes of the 19 bioclimatic variables used in this study follow those of Worldclim and ANUCLIM.

**TABLE S3** Non-synonymous (dN) and synonymous (dS) substitutions for *R. ferrumequinum*.

**TABLE S4** Genetic diversity values for the MHC II-DRB exon 2 locus and microsatellite within populations of *R. ferrumequinum*.

**TABLE S5** Genetic diversity values for the MHC II-DRB exon 2 locus and microsatellite in genetic lineages of *R. ferrumequinum*.

**FIGURE S1.** (A) BIC values across the number of clusters using the “find. clusters” function in the R package “adegenet”. K = 6 had the lowest BIC values. (B) Output from discriminant analysis of principal components (DAPC) based on physiochemical properties of peptide-interacting codons showing the four recovered MHC supertypes.

**FIGURE S2**. ρ^2^ values were calculated using the Hmisc package “varclus” function in the R. (A) MHC variations; (B) climatic factors.

**FIGURE S3.** Amino acid alignment of MHC class II-*DRB* alleles characterized in *R. ferrumequinum*.

**FIGURE S4.** Alignment of MHC class II-*DRB* alleles characterized in *R. ferrumequinum*. Dots mark identity with the top sequence. *Signify the amino-acid positions of ABS and conserved sites of the human HLA-DR1 β-chain (Brown et al., 1993; Stern et al., 1994). Species-specific positive selected sites identified by DATAMONKEY are indicated by two models: FUBAR and FEL.

**FIGURE S5.** Scatterplot of discriminant analysis of principal components (DAPC) of *R. ferrumequinum* for the genetic population structures and genetic lineages structures based on the MHC-DRB locus (A, B) and microsatellites (C, D). The inset shows the discriminant analysis (DA) eigenvalues.

**Text S1 Supplemental Methods – 2-step PCR description**

**TABLE S1** Sampling information of *R. ferrumequinum* used in this study.

| Sample | Number of alleles | Raw_num | Mean_len | Clean_num | Mean_len | Individuals consistent with microsatellite data |
| --- | --- | --- | --- | --- | --- | --- |
| BJ01 | 4 | 92764 | 450.86 | 91721 | 410.97 | √ |
| BJ02 | 3 | 41583 | 420.06 | 41123 | 381.36 | √ |
| BJ03 | 5 | 100025 | 437.8 | 99196 | 397.19 | √ |
| BJ04 | 1 | 42665 | 413.57 | 41724 | 376.86 | √ |
| BJ05 | 4 | 140754 | 431.07 | 139883 | 387.64 | √ |
| BJ07 | 2 | 37860 | 443.68 | 37440 | 402.79 |  |
| BJ08 | 5 | 116762 | 433.95 | 116108 | 390.2 |  |
| BJ09 | 6 | 32024 | 449.11 | 31703 | 405.23 | √ |
| BJ10 | 6 | 37210 | 449.55 | 36912 | 407.07 |  |
| FD01 | 5 | 99562 | 455.09 | 99157 | 412.26 | √ |
| FD02 | 5 | 100550 | 452.63 | 100136 | 409.76 | √ |
| FD03 | 3 | 91548 | 452.27 | 91235 | 409.52 | √ |
| FD04 | 3 | 85536 | 450.37 | 85178 | 407.36 | √ |
| FD05 | 4 | 81134 | 451.24 | 80794 | 409.56 | √ |
| FD06 | 3 | 104204 | 458.25 | 103860 | 415.5 | √ |
| GS01 | 3 | 33661 | 453.42 | 33415 | 409.78 | √ |
| GS02 | 4 | 165756 | 433.36 | 164547 | 392.99 | √ |
| GS03 | 3 | 37908 | 446.07 | 37619 | 403.56 | √ |
| GS04 | 3 | 37386 | 455.91 | 37197 | 412.47 | √ |
| GS05 | 4 | 37146 | 452.7 | 36918 | 409.21 | √ |
| GS06 | 6 | 146471 | 439.31 | 145445 | 395.84 | √ |
| GS07 | 5 | 129048 | 432.01 | 128361 | 389.8 | √ |
| GS08 | 3 | 151779 | 460.47 | 151263 | 417.24 | √ |
| GS09 | 3 | 150597 | 414.13 | 149670 | 372.09 | √ |
| GS10 | 2 | 33766 | 439.84 | 33512 | 396.7 | √ |
| HeN033 | 4 | 26389 | 452.28 | 26180 | 407.1 | √ |
| HeN034 | 4 | 19824 | 455.19 | 19733 | 412.24 | √ |
| HeN035 | 5 | 40366 | 455.65 | 40253 | 413.02 | √ |
| HeN036 | 4 | 33911 | 449.09 | 33770 | 406.73 | √ |
| HeN037 | 2 | 56886 | 450.19 | 56682 | 407.89 | √ |
| HeN038 | 4 | 43631 | 455.78 | 43488 | 413.49 | √ |
| HeN039 | 5 | 54370 | 453.45 | 54018 | 409.05 | √ |
| HeN040 | 2 | 44521 | 450.39 | 44108 | 403.82 | √ |
| HeN041 | 2 | 40946 | 452.37 | 40731 | 409.13 | √ |
| HeN042 | 4 | 134647 | 424.24 | 134004 | 383.23 |  |
| HY01 | 4 | 41292 | 449.87 | 40995 | 409.07 | √ |
| HY02 | 4 | 59019 | 449.28 | 58756 | 407.17 | √ |
| HY03 | 2 | 62072 | 448.85 | 61386 | 404.19 | √ |
| HY04 | 4 | 37440 | 450.71 | 37069 | 410.12 | √ |
| HY05 | 3 | 53709 | 450.79 | 53193 | 403.82 | √ |
| HY06 | 3 | 56494 | 447.2 | 56191 | 406.85 | √ |
| HY07 | 2 | 38866 | 442.11 | 38297 | 401.22 | √ |
| HY08 | 4 | 39247 | 441.37 | 38945 | 399.89 | √ |
| HY09 | 2 | 56149 | 452.67 | 55856 | 410.55 | √ |
| HY10 | 2 | 110078 | 453.35 | 109559 | 411.19 | √ |
| JA01 | 5 | 59127 | 459.88 | 58872 | 415 | √ |
| JA02 | 2 | 78645 | 422.66 | 77818 | 379.43 | √ |
| JA03 | 3 | 84935 | 436.34 | 84427 | 391.64 |  |
| JA04 | 4 | 34806 | 452.82 | 34476 | 411.37 | √ |
| JA05 | 1 | 30401 | 435.05 | 29711 | 401.26 | √ |
| JA06 | 2 | 64132 | 425.1 | 61467 | 397.85 | √ |
| JA07 | 4 | 94732 | 442.1 | 94169 | 400 |  |
| JA08 | 4 | 98342 | 417.09 | 97631 | 375.8 |  |
| JL201 | 5 | 117043 | 420.14 | 116333 | 378.26 | √ |
| JL202 | 3 | 118447 | 407.86 | 117244 | 365.57 | √ |
| JL203 | 4 | 126373 | 418.45 | 125282 | 374.25 | √ |
| JL204 | 4 | 102565 | 416.95 | 101804 | 376.43 | √ |
| JL205 | 6 | 81268 | 435.48 | 80501 | 395.42 | √ |
| JL2838 | 6 | 93802 | 416.16 | 92911 | 375.49 | √ |
| JL206 | 5 | 110867 | 428.25 | 110004 | 387.24 |  |
| JL207 | 4 | 68748 | 461.63 | 68556 | 419.32 | √ |
| JL208 | 4 | 94044 | 412.49 | 93141 | 371.88 | √ |
| JL209 | 6 | 88393 | 453.79 | 88128 | 411.14 | √ |
| JL210 | 5 | 63540 | 451.05 | 63325 | 407.17 |  |
| JL211 | 6 | 26535 | 453.76 | 26430 | 410.97 |  |
| SD01 | 4 | 45037 | 458.77 | 44852 | 415.74 | √ |
| SD02 | 3 | 141063 | 425.73 | 140476 | 383.5 | √ |
| SD03 | 4 | 133351 | 456.05 | 132743 | 412.59 | √ |
| SD04 | 6 | 89378 | 451.32 | 87970 | 412.96 | √ |
| SD05 | 3 | 142755 | 426.48 | 141970 | 383.88 | √ |
| SD06 | 2 | 95583 | 405.4 | 95218 | 363.51 | √ |
| SD07 | 1 | 143623 | 328.7 | 139802 | 291.91 | √ |
| SD08 | 4 | 41019 | 452.67 | 40781 | 411.47 | √ |
| SD09 | 4 | 70443 | 461.96 | 70074 | 420.4 | √ |
| SD10 | 3 | 118056 | 451.45 | 117563 | 408.91 | √ |
| SD29 | 3 | 38013 | 446.97 | 37696 | 401.46 | √ |
| SD44 | 6 | 38230 | 457.36 | 38093 | 414.44 | √ |
| SD54 | 4 | 41944 | 454.44 | 41792 | 411.83 | √ |
| SD55 | 4 | 46480 | 445.8 | 46144 | 404.21 | √ |
| SD56 | 6 | 121688 | 445.97 | 121107 | 403.09 | √ |
| SD59 | 6 | 36352 | 457.74 | 36172 | 415.27 | √ |
| SD64 | 2 | 30397 | 456.95 | 30219 | 415.02 | √ |
| SD67 | 3 | 45012 | 451.58 | 44739 | 409.08 | √ |
| SX01 | 1 | 55231 | 454.54 | 54687 | 407.97 | √ |
| SX02 | 4 | 91602 | 454.02 | 90930 | 409 | √ |
| SX03 | 3 | 102344 | 448.66 | 101513 | 403.88 | √ |
| SX04 | 3 | 72720 | 444 | 72159 | 397.4 | √ |
| SX06 | 4 | 24754 | 448.83 | 24554 | 407.77 | √ |
| SX07 | 3 | 69882 | 446.38 | 69193 | 403.76 | √ |
| SX31 | 4 | 89513 | 447.36 | 88966 | 402.98 | √ |
| SX32 | 4 | 41958 | 456.73 | 41756 | 414.09 | √ |
| YN01 | 1 | 79335 | 400.58 | 78451 | 360.77 | √ |
| YN02 | 3 | 128968 | 431.81 | 128193 | 389.97 | √ |
| YN157 | 4 | 72912 | 451.13 | 72559 | 406.81 | √ |
| YN177 | 3 | 128622 | 418.12 | 126606 | 373.18 | √ |
| YN202 | 5 | 89452 | 451.56 | 88993 | 407.81 | √ |
| YN204 | 5 | 65418 | 457.53 | 65079 | 412.02 | √ |
| YN205 | 7 | 72344 | 453.73 | 72020 | 410.62 | √ |
| YN206 | 4 | 125742 | 428.9 | 124517 | 387.78 | √ |
| YN207 | 1 | 81241 | 419.74 | 80288 | 375.37 | √ |
| YN53 | 3 | 45860 | 431.24 | 44938 | 392.4 | √ |
| ZJ01 | 4 | 80168 | 454.51 | 79874 | 411.7 | √ |
| ZJ02 | 3 | 94621 | 458.32 | 94231 | 414.92 | √ |
| ZJ03 | 4 | 145375 | 446.09 | 144775 | 403.35 | √ |
| ZJ04 | 5 | 155730 | 453.43 | 154994 | 410.33 | √ |
| JL301 | 4 | 47749 | 442.98 | 46718 | 404.53 | √ |
| JL302 | 1 | 53611 | 449.65 | 53113 | 403.82 | √ |
| JL303 | 5 | 87641 | 441.55 | 87196 | 396.54 | √ |
| JL304 | 5 | 67710 | 424.52 | 67257 | 380.34 | √ |
| JL305 | 3 | 55434 | 437.76 | 53500 | 403.17 |  |
| JL306 | 3 | 55334 | 452.73 | 54913 | 407.46 | √ |
| JL307 | 2 | 42950 | 435.39 | 41069 | 406.44 | √ |
| JL308 | 2 | 63907 | 427.62 | 63473 | 383.66 | √ |
| JL309 | 2 | 68680 | 412.18 | 68215 | 369.95 |  |
| JL310 | 1 | 38726 | 422.91 | 35845 | 404.15 | √ |
| 191Y | 3 | 53330 | 431.09 | 52688 | 389.3 | √ |
| 197Y | 1 | 73228 | 381.16 | 72523 | 339.55 | √ |
| 200Y | 1 | 54140 | 425.47 | 53150 | 385.01 | √ |
| 242Y | 4 | 63136 | 436.74 | 62379 | 395.23 | √ |
| 249Y | 3 | 53174 | 441.31 | 52584 | 399.15 | √ |
| 257Y | 3 | 93520 | 424.78 | 92348 | 383.82 | √ |

**TABLE S2.** The codes of the 19 bioclimatic variables used in this study, which followed those of Worldclim and ANUCLIM.

| **Abbreviation** | **Bioclimatic Variable** |
| --- | --- |
| BIO1 | Annual Mean Temperature |
| BIO2 | Monthly Mean Temperature |
| BIO3 | Isothermality (Bio2/Bio7) (* 100) |
| BIO4 | Temperature Seasonality (standard deviation *100) |
| BIO5 | Max Temperature of Warmest Month |
| BIO6 | Min Temperature of Coldest Month |
| BIO7 | Temperature Annual Range (Bio5-Bio6) |
| BIO8 | Mean Temperature of Wettest Quarter |
| BIO9 | Mean Temperature of Driest Quarter |
| BIO10 | Mean Temperature of Warmest Quarter |
| BIO11 | Mean Temperature of Coldest Quarter |
| BIO12 | Annual Precipitation |
| BIO13 | Precipitation of Wettest Month |
| BIO14 | Precipitation of Driest Month |
| BIO15 | Precipitation Seasonality (Coefficient of Variation) |
| BIO16 | Precipitation of Wettest Quarter |
| BIO17 | Precipitation of Driest Quarter |
| BIO18 | Precipitation of Warmest Quarter |
| BIO19 | Precipitation of Coldest Quarter |

**TABLE S3.** Non-synonymous (dN) and synonymous (dS) substitutions for *R. ferrumequinum*.

|  | Non-ABS |  |  |  | ABS |  |  |  |
| --- | --- | --- | --- | --- | --- | --- | --- | --- |
| Populations | dS | dN | Z | p | dS | dN | Z | p |
| HeN | 0.037 | 0.076 | 2.452 | 0.016 | 0.063 | 0.245 | 5.42 | 0 |
| BJ | 0.037 | 0.077 | 1.926 | 0.056 | 0.053 | 0.267 | 4.725 | 0 |
| FD | 0.039 | 0.085 | 2.815 | 0.006 | 0.085 | 0.263 | 3.94 | 0 |
| HY | 0.034 | 0.084 | 2.59 | 0.01 | 0.051 | 0.277 | 5.1 | 0 |
| JL1 | 0.021 | 0.076 | 3.27 | 0 | 0.036 | 0.251 | 4.65 | 0 |
| JL3 | 0.035 | 0.079 | 2.23 | 0.03 | 0.049 | 0.229 | 4.85 | 0 |
| SD | 0.029 | 0.071 | 2.69 | 0.01 | 0.029 | 0.071 | 2.678 | 0.008 |
| SX | 0.028 | 0.073 | 2.72 | 0.01 | 0.028 | 0.073 | 2.665 | 0.009 |
| JL3 | 0.037 | 0.083 | 2.353 | 0.02 | 0.037 | 0.083 | 2.432 | 0.016 |
| YN | 0.044 | 0.106 | 3.22 | 0 | 0.044 | 0.106 | 3.33 | 0 |
| ZJ | 0.047 | 0.07 | 1.06 | 0.291 | 0.067 | 0.259 | 4.01 | 0 |
| GS | 0.014 | 0.031 | 1.52 | 0.131 | 0.06 | 0.24 | 4.73 | 0 |
| ALL | 0.028 | 0.034 | 0.35 | 0.72 | 0.055 | 0.253 | 5.22 | 0 |

| region | π | P | h | AR | NaI | ST1 | ST2 | ST3 | ST4 | ST5 | ST6 | ST_SUM | SSR_Ho_ | SSR_He_ |
| --- | --- | --- | --- | --- | --- | --- | --- | --- | --- | --- | --- | --- | --- | --- |
| CE | 0.062 | 18 | 31 | 1.504 | 7.322 | 14 | 4 | 55 | 27 | 9 | 10 | 119 | 0.393 | 0.639 |
| NE | 0.065 | 8 | 17 | 1.391 | 6.529 | 14 | 0 | 22 | 17 | 1 | 0 | 54 | 0.444 | 0.522 |
| SW | 0.075 | 19 | 29 | 1.717 | 3.275 | 15 | 1 | 22 | 8 | 4 | 4 | 54 | 0.299 | 0.632 |

**TABLE S4.** Genetic diversity values for the MHC II-DRB exon 2 locus and microsatellite within populations of *R. ferrumequinum*.

Genetic diversity values for MHC II-DRB exon 2: N = sample size; P = private allele; S = number of segregating sites; π = average nucleotide diversity; h = number of haplotypes; AR = allele richness; NaI = mean number of alleles per individual; MHC functional supertypes values: ST_fre = supertype frequency; ST_SUM = total number of supertype. Genetic diversity values for microsatellite: SSR_Ho_ = observe heterozygosity; SSR_He_ = expected heterozygosity.

| Group | Region | N | P | S | π | h | NaI | AR | ST1 | ST2 | ST3 | ST4 | ST5 | ST6 | ST_SUM | SSR_AR_ | SSR_Ho_ | SSR_He_ |
| --- | --- | --- | --- | --- | --- | --- | --- | --- | --- | --- | --- | --- | --- | --- | --- | --- | --- | --- |
| BJ | CE | 9 | 2 | 48 | 0.063 | 12 | 3 | 1.090 | 4 | 1 | 7 | 2 | 1 | 4 | 16 | 2.765 | 3 | 2.751 |
| HeN | CE | 16 | 3 | 55 | 0.062 | 11 | 4.636 | 1.068 | 1 | 2 | 10 | 4 | 4 | 3 | 25 | 3.573 | 4.429 | 3.684 |
| HY | CE | 10 | 1 | 58 | 0.069 | 12 | 2.5 | 1.096 | 4 | 0 | 9 | 8 | 1 | 1 | 20 | 3.405 | 4.571 | 3.467 |
| SD | CE | 18 | 4 | 55 | 0.043 | 12 | 5.667 | 1.079 | 4 | 1 | 17 | 7 | 0 | 1 | 31 | 3.444 | 4.286 | 3.397 |
| SX | CE | 8 | 0 | 47 | 0.059 | 7 | 3.714 | 1.079 | 1 | 0 | 8 | 2 | 3 | 0 | 15 | 3.505 | 4 | 3.187 |
| ZJ | CE | 4 | 2 | 37 | 0.06 | 6 | 2.667 | 1.094 | 0 | 0 | 4 | 4 | 0 | 1 | 6 | 2.952 | 3.143 | 2.473 |
| JL1 | NE | 8 | 0 | 45 | 0.061 | 10 | 2.5 | 1.085 | 3 | 0 | 2 | 6 | 1 | 0 | 13 | 2.595 | 2.857 | 2.197 |
| JL2 | NE | 12 | 1 | 47 | 0.063 | 13 | 4.462 | 1.111 | 8 | 0 | 12 | 5 | 0 | 0 | 31 | 2.743 | 3.143 | 2.349 |
| JL3 | NE | 10 | 0 | 41 | 0.067 | 6 | 4.667 | 1.070 | 3 | 0 | 8 | 6 | 0 | 0 | 18 | 2.429 | 2.429 | 2.128 |
| YN | SW | 10 | 8 | 74 | 0.084 | 14 | 2.571 | 1.118 | 7 | 1 | 7 | 2 | 1 | 1 | 21 | 3.326 | 4.143 | 3.174 |
| FD | SW | 6 | 5 | 53 | 0.071 | 13 | 1.769 | 1.149 | 3 | 0 | 6 | 3 | 1 | 2 | 11 | 3.601 | 4.143 | 3.274 |
| GS | SW | 10 | 4 | 57 | 0.063 | 11 | 3.273 | 1.095 | 5 | 0 | 9 | 3 | 2 | 1 | 22 | 2.969 | 3.429 | 2.712 |

**TABLE S5.** Genetic diversity values for the MHC II-DRB exon 2 locus and microsatellite in genetic lineages of *R. ferrumequinum*.

Genetic diversity values for MHC II-DRB exon 2: N = sample size; P = private allele; S = number of segregating sites; π = average nucleotide diversity; h = number of haplotypes; NaI = mean number of alleles per individual; AR = allelic richness. MHC functional supertypes values: ST_fre = supertype frequency; ST_SUM = total number of supertypes. Genetic diversity values for microsatellite: SSR_AR_ = allelic richness; SSR_Ho_ = observe heterozygosity; SSR_He_ = expected heterozygosity.

**TABLE S6.** The supertype groups to which the MHC alleles belongs.

| Allele | ST | Allele | ST | Allele | ST |
| --- | --- | --- | --- | --- | --- |
| Rhfe01 | 3 | Rhfe21 | 3 | Rhfe40 | 3 |
| Rhfe02 | 4 | Rhfe22 | 4 | Rhfe41 | 4 |
| Rhfe03 | 6 | Rhfe23 | 3 | Rhfe42 | 5 |
| Rhfe04 | 5 | Rhfe24 | 5 | Rhfe43 | 3 |
| Rhfe05 | 1 | Rhfe25 | 4 | Rhfe44 | 3 |
| Rhfe06 | 6 | Rhfe26 | 6 | Rhfe45 | 1 |
| Rhfe07 | 2 | Rhfe27 | 1 | Rhfe46 | 4 |
| Rhfe08 | 1 | Rhfe28 | 4 | Rhfe47 | 1 |
| Rhfe09 | 1 | Rhfe29 | 3 | Rhfe48 | 4 |
| Rhfe10 | 4 | Rhfe30 | 1 | Rhfe50 | 5 |
| Rhfe11 | 1 | Rhfe31 | 4 | Rhfe51 | 4 |
| Rhfe12 | 4 | Rhfe32 | 6 | Rhfe52 | 1 |
| Rhfe13 | 3 | Rhfe33 | 4 | Rhfe53 | 1 |
| Rhfe14 | 5 | Rhfe34 | 4 | Rhfe54 | 3 |
| Rhfe15 | 6 | Rhfe35 | 4 | Rhfe55 | 4 |
| Rhfe16 | 1 | Rhfe36 | 1 | Rhfe56 | 6 |
| Rhfe17 | 3 | Rhfe37 | 4 | Rhfe57 | 2 |
| Rhfe18 | 6 | Rhfe38 | 4 | Rhfe58 | 3 |
| Rhfe20 | 3 | Rhfe39 | 3 | Rhfe59 | 4 |

**FIGURE S1** (A): BIC values across number of clusters using the “find.clusters” function in the R package “adegenet”. K = 6 had the lowest BIC values. (B): Output from discriminant analysis of principal components (DAPC) based on physiochemical properties of peptide-interacting codons showing the four recovered MHC supertypes.


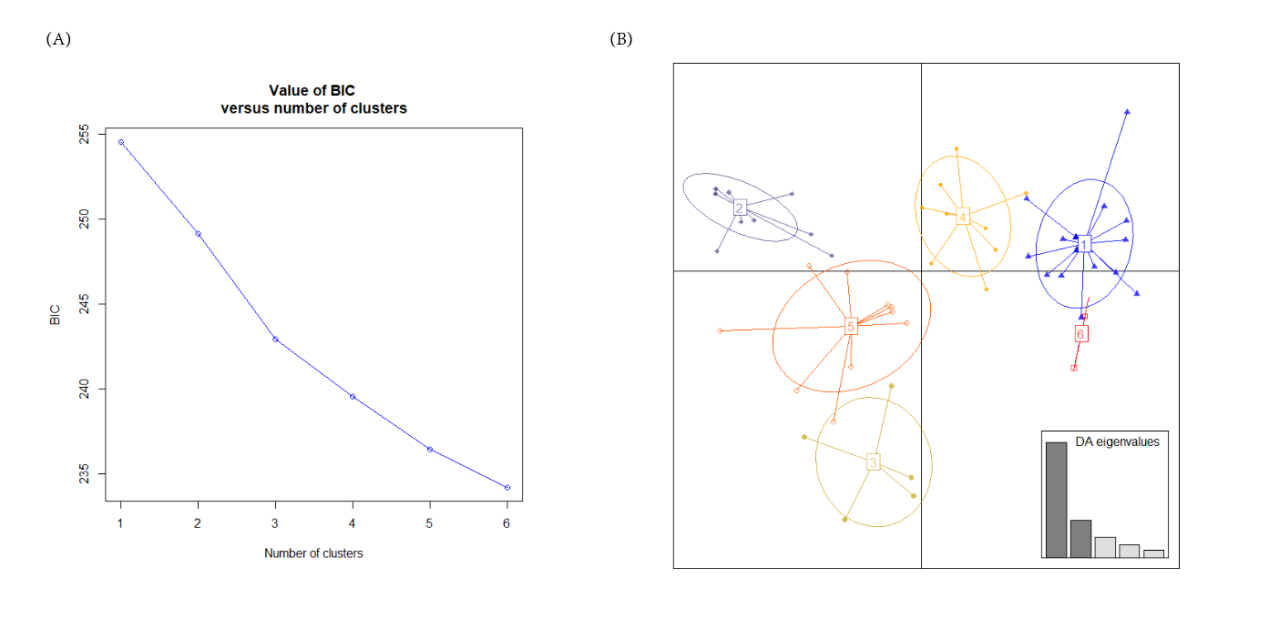


**FIGURE S2** ρ^2^ values were calculated using the Hmisc package “varclus” function in the R. (A) climatic factors; (B) MHC variations.


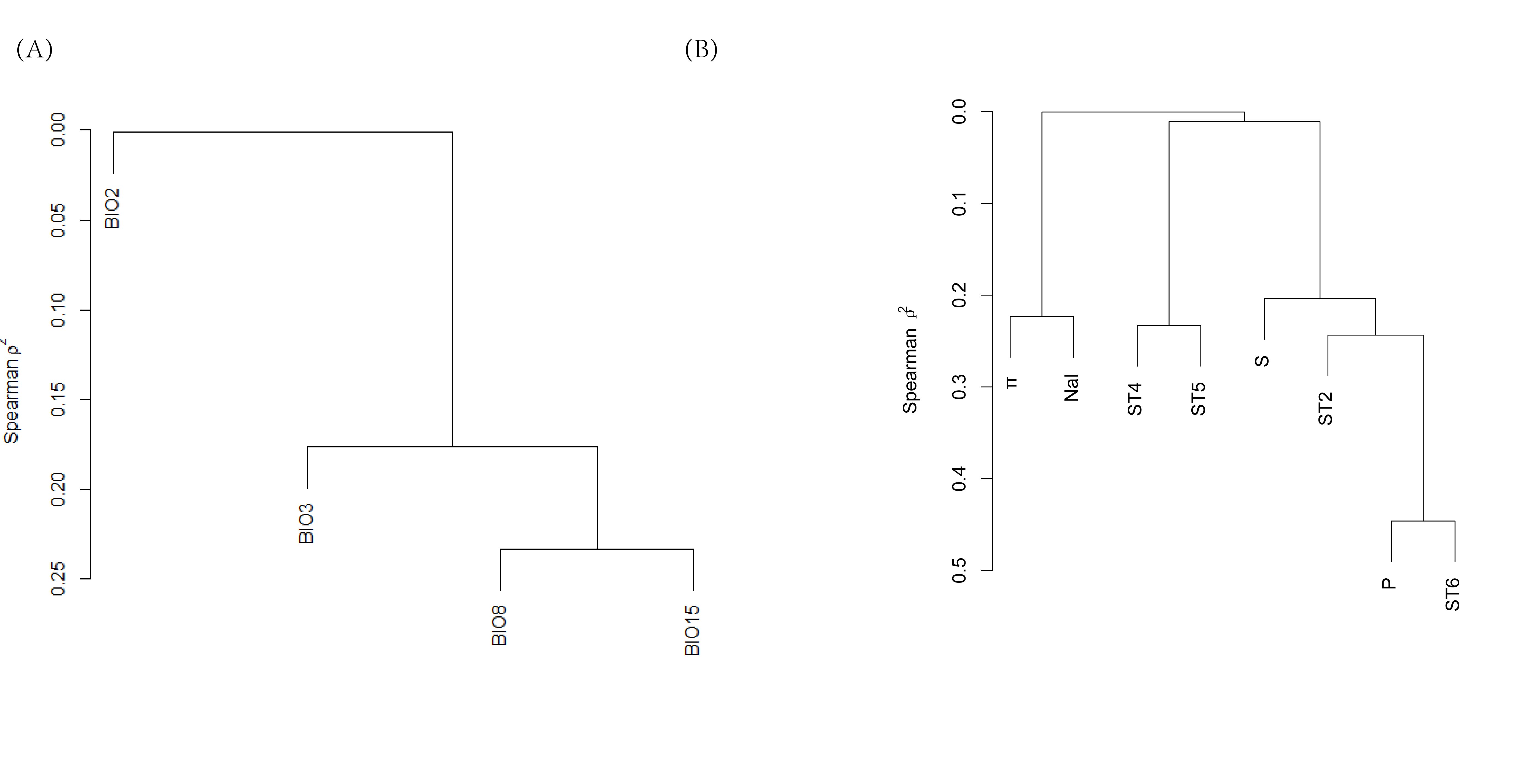


**FIGURE S3** Nucleotide alignment of MHC II-DRB exon 2 alleles from *R. ferrumequinum.*


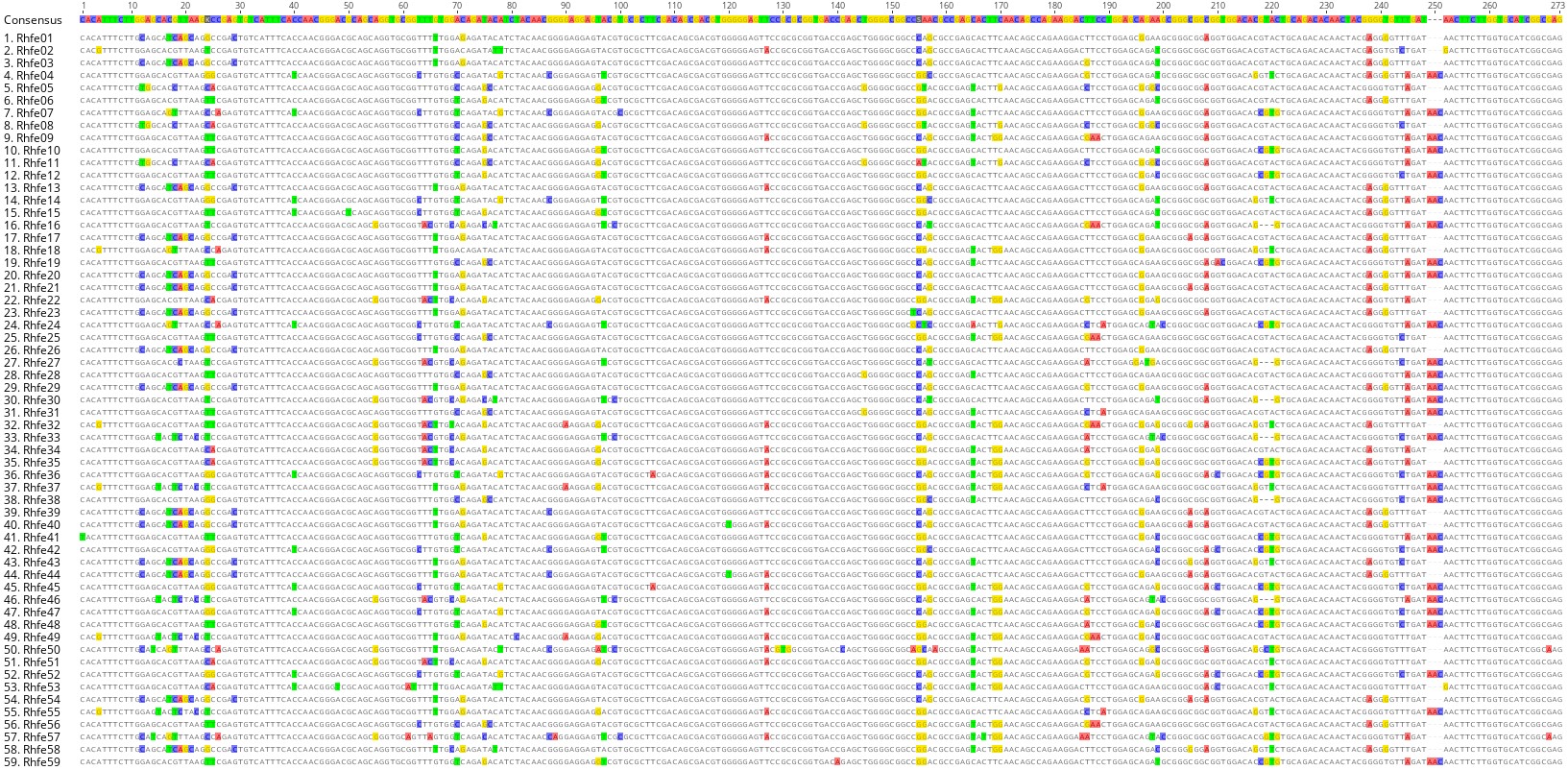


**FIGURE S4** Scatterplot of discriminant analysis of principal components (DAPC) of *R. ferrumequinum* for the genetic population structures and genetic lineages structures based on the MHC-DRB locus (A, B) and microsatellites (C, D). The inset shows the discriminant analysis (DA) eigenvalues.


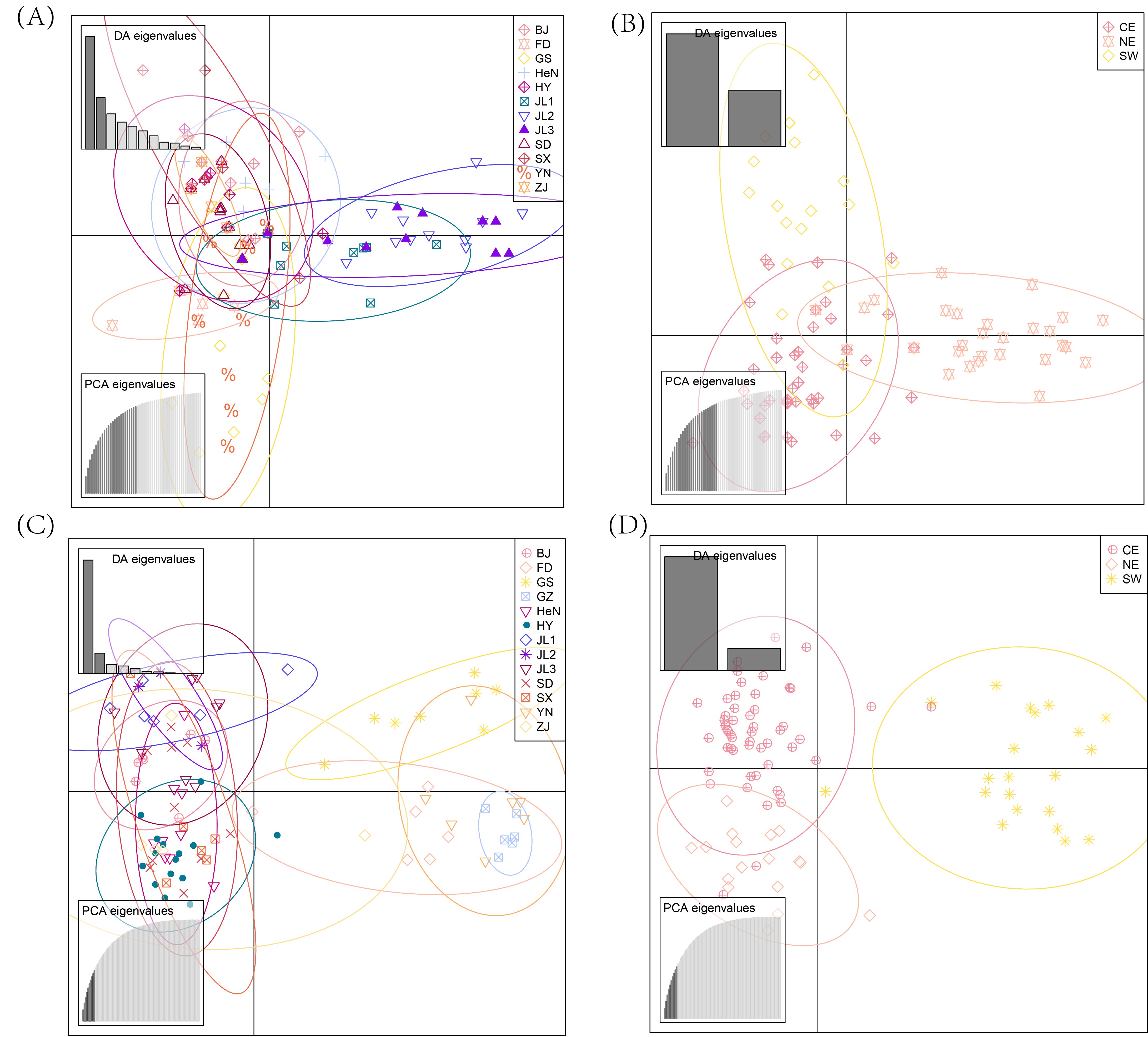


**FIGURE S5** Amino acid alignment of MHC class II-*DRB* alleles characterized in *R. ferrumequinum*. Dots mark identity with the top sequence. *Signify the amino-acid positions of ABS and conserved sites of the human HLA-DR1 β-chain (Brown et al., 1993; Stern et al., 1994). Species-specific positive selected sites identified by DATAMONKEY are indicated by two models: FUBAR and FEL.


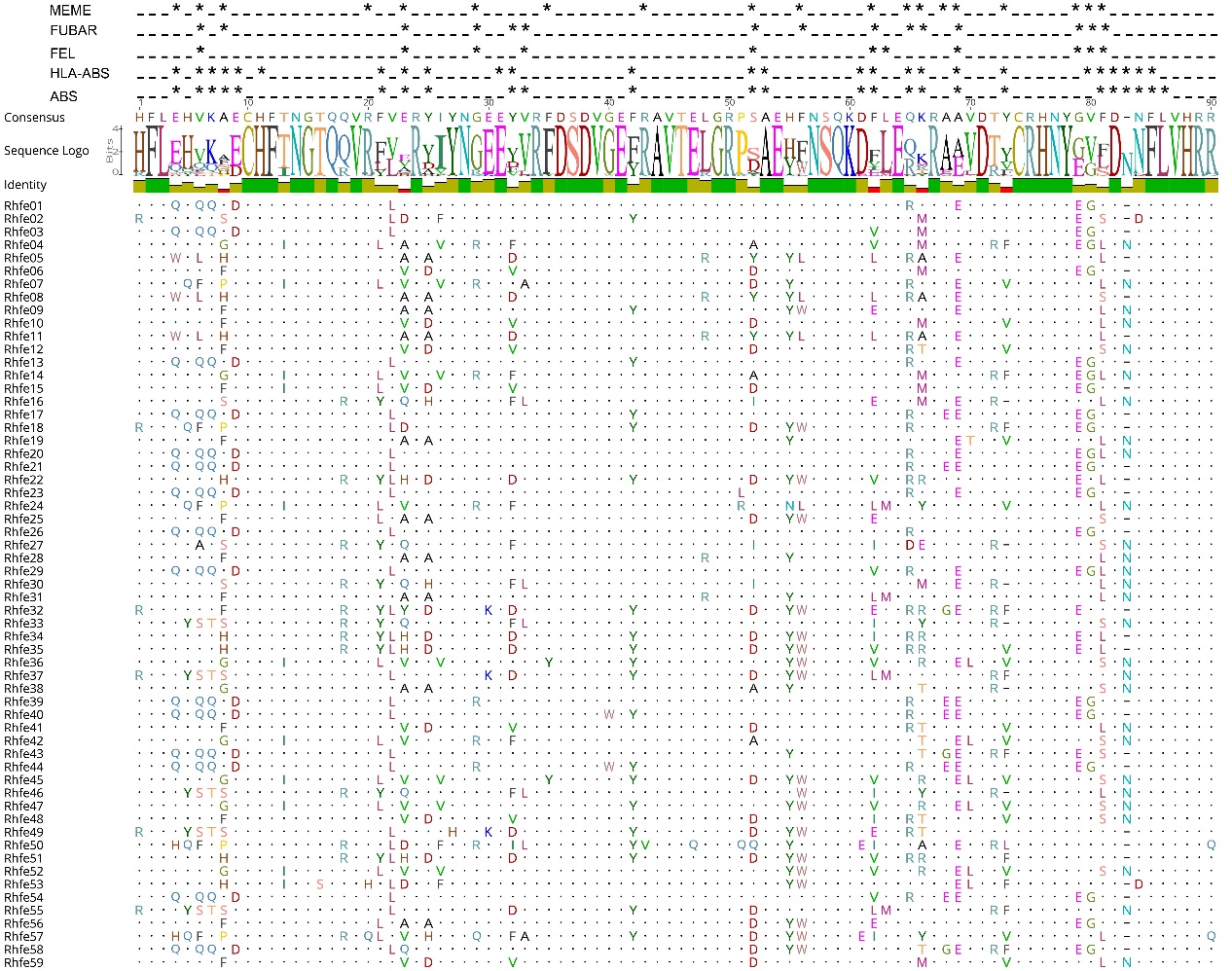


**Text S1 Supplemental Methods – 2-step PCR description**

The first PCR step was performed using the primers 0702-1F and 0702-1R. PCR amplifications were performed with a Mastercycler Nexus GSX1 (Eppendorf, Germany) in a final volume of 30 μl containing1 μl Bar-PCR primer F and 1 μl primer R, 10 ng of genomic DNA, and 15 μl 2× Taq Master Mix. Thermocycling started with an initial denaturation step of 94°C for 3 min; with five cycles at 94°C for 30 s, 45°C for 20 s, 65°C for 30 s, 20 cycles of denaturation at 94°C for 20 s, annealing at 55°C for 20 s, extension at 72°C for 30 s, and a final extension at 72°C for 5 min. Another 30 μl reaction mixture contained 20 ng of the first step amplification product, 15 μl 2× Taq master Mix, and 1 μl of each primer was used for the second PCR step under the following conditions: denaturation at 95°C for 3 min, five cycles of denaturation at 94°C for 20 s, annealing at 55°C for 20 s and extension at 72°C for 30 s, and a final extension at 72°C for 5 min. PCR amplicons were purified with Agencourt AMPure XP (Beckman Coulter, USA) and DNA concentration of each sample was quantified using the Qubit 2.0 DNA Assay Kit (Life, USA) to normalize sample.
